# A combined analysis of immunogenicity, antibody kinetics and vaccine efficacy from phase 2 trials of the RTS,S malaria vaccine

**DOI:** 10.1186/s12916-014-0117-2

**Published:** 2014-07-10

**Authors:** Michael T White, Philip Bejon, Ally Olotu, Jamie T Griffin, Kalifa Bojang, John Lusingu, Nahya Salim, Salim Abdulla, Nekoye Otsyula, Selidji T Agnandji, Bertrand Lell, Kwaku Poku Asante, Seth Owusu-Agyei, Emmanuel Mahama, Tsiri Agbenyega, Daniel Ansong, Jahit Sacarlal, John J Aponte, Azra C Ghani

**Affiliations:** 1 MRC Centre for Outbreak Analysis and Modelling, Department of Infectious Disease Epidemiology, Imperial College London, London W2 1PG, UK; 2 KEMRI-Wellcome Trust Research Programme, Kenya Medical Research Institute, Kilifi, Kenya; 3 Centre for Clinical Vaccinology and Tropical Medicine, University of Oxford, Oxford, UK; 4 Medical Research Council Unit, Fajara, The Gambia; 5 National Institute for Medical Research, Tanga Centre, Tanga, Tanzania; 6 Ifakara Health Institute, Bagamoyo, Tanzania; 7 Kenya Medical Research Institute, and US Army Medical Research Unit–Kenya, Nairobi, Kenya; 8 Medical Research Unit, Albert Schweitzer Hospital, Lambaréné, Gabon; 9 Institute of Tropical Medicine, University of Tübingen, Tübingen, Germany; 10 Kintampo Health Research Centre, Kintampo, Ghana; 11 School of Medical Sciences, Kumasi, Ghana; 12 Centro de Investigação em Saúde de Manhiça, Manhiça, Mozambique; 13 Faculdade de Medicina, Universidade Eduardo Mondlane, Avenida do Zimbabwe, Maputo, Mozambique; 14 Barcelona Centre for International Health Research (CRESIB), Universitat de Barcelona, Barcelona, Spain

**Keywords:** Malaria, Vaccine, Circumsporozoite protein, Antibody, RTS,S, Phase 2 clinical trials, Mathematical model, Clinical immunity

## Abstract

**Background:**

The RTS,S malaria vaccine is currently undergoing phase 3 trials. High vaccine-induced antibody titres to the circumsporozoite protein (CSP) antigen have been associated with protection from infection and episodes of clinical malaria.

**Methods:**

Using data from 5,144 participants in nine phase 2 trials, we explore predictors of vaccine immunogenicity (anti-CSP antibody titres), decay in antibody titres, and the association between antibody titres and clinical outcomes. We use empirically-observed relationships between these factors to predict vaccine efficacy in a range of scenarios.

**Results:**

Vaccine-induced anti-CSP antibody titres were significantly associated with age (*P* = 0.04), adjuvant (*P* <0.001), pre-vaccination anti-hepatitis B surface antigen titres (*P* = 0.005) and pre-vaccination anti-CSP titres (*P* <0.001). Co-administration with other vaccines reduced anti-CSP antibody titres although not significantly (*P* = 0.095). Antibody titres showed a bi-phasic decay over time with an initial rapid decay in the first three months and a second slower decay over the next three to four years. Antibody titres were significantly associated with protection, with a titre of 51 (95% Credible Interval (CrI): 29 to 85) ELISA units/ml (EU/mL) predicted to prevent 50% of infections in children. Vaccine efficacy was predicted to decline to zero over four years in a setting with entomological inoculation rate (EIR) = 20 infectious bites per year (ibpy). Over a five-year follow-up period at an EIR = 20 ibpy, we predict RTS,S will avert 1,782 cases per 1,000 vaccinated children, 1,452 cases per 1,000 vaccinated infants, and 887 cases per 1,000 infants when co-administered with expanded programme on immunisation (EPI) vaccines. Our main study limitations include an absence of vaccine-induced cellular immune responses and short duration of follow-up in some individuals.

**Conclusions:**

Vaccine-induced anti-CSP antibody titres and transmission intensity can explain variations in observed vaccine efficacy.

## Background

The candidate malaria vaccine RTS,S/AS01 is currently in phase 3 trials in multiple sites across sub-Saharan Africa [[1],[2]]. Efficacy against clinical malaria over one year of follow-up was 55.8% (97.5% confidence interval (CI): 50.6% to 60.4%) in children 5- to 17-months old [[1]] but was significantly lower in infants 6- to 12-weeks old (31.3%, 97.5% CI: 23.6% to 38.3%) [[2]]. Vaccine-induced geometric mean anti-circumsporozoite protein (CSP) antibody titres following the third dose in the 6- to 12-week cohort was 209 ELISA units/ml (EU/mL) (95% CI: 197 to 222) [[2]] significantly lower than in the 5- to 17-month cohort (621 EU/mL, 95% CI: 592 to 652) [[1]]. Two potential reasons for the lower observed vaccine efficacy in the 6- to 12-week cohort have been proposed: (1) that co-administration with other expanded programme on immunisation (EPI) vaccines in the 6- to 12-week cohort may have interfered with the response to the RTS,S vaccine; and (2) that the 6- to 12-week cohort have reduced ability to mount a sustained and effective immune response [[3]], perhaps due to interference from maternal antibodies. Substantial variation in measured vaccine efficacy was also observed in the phase 2 trials, with efficacy demonstrated to depend on transmission intensity, choice of adjuvant and age at vaccination [[4]]. However, to date, the extent to which this variation can be explained by vaccine-induced anti-CSP antibody titres has not been explored.

Here, using data from nine phase 2 trials of the RTS,S vaccine in ten different trial sites, we investigate the association between vaccine-induced anti-CSP antibodies, their decay over time and vaccine efficacy against parasitological infection. We combine this data with a previously published model for the acquisition of clinical immunity [[5]] to account for reductions in efficacy against clinical malaria due to lower levels of naturally-acquired immunity in vaccinated compared to unvaccinated children. These results from heterogeneous groups in the phase 2 trials will complement the critical analyses from the multi-site phase 3 trial [[1],[2]].

## Methods

### Data

Phase 2 trials of the RTS,S vaccine were identified from GlaxoSmithKline Vaccines’ registry of trials and the individual-level data were provided by GSK Vaccines. Characteristics of the trials, undertaken at ten sites in six African countries, are summarised in Table 1. Healthy adults, children or infants were recruited after clinical and laboratory screening to exclude participants with clinically significant disease. Five trials considered infection as an endpoint implementing active case detection for *Plasmodium falciparum* infection (ACDi). Three trials used episodes of clinical malaria as an endpoint, one using active case detection for clinical malaria (ACDc) [[6]], and two using passive case detection (PCD) for clinical malaria [[7],[8]]. One trial considered both ACDi and PCD for clinical malaria [[7]]. Two additional trials monitored immunogenicity but did not follow-up for clinical endpoints [[9],[10]]. RTS,S was co-administered with other vaccines in two trials [[8],[11]]. In total, we analysed data from 5,144 trial participants. All trials received ethical approval from relevant local ethics committees. Information on the ethical approval regarding the trials including in this analysis can be found in Additional file 1.

**Table 1:** Characteristics of phase 2 trial sites

**Site**	**Participants (RTS,S)**	**Active vaccine**	**Median age (IQR)**	**Parasite prevalence**^**a**^	**Schedule**	**Peak anti-CSP titre (95% range)**
Gambia [[12]]	250 (136)	RTS,S/AS02A	24 (19 to 34) years	70%	0,1,5,14 months	25 (13 to 43) μg/mL
Kisumu, Kenya [[13]]	250 (159)	RTS,S/AS02A and RTS,S/AS01B	25 (21 to 29) years	60%	0,1,2 months	34 (2 to 210) EU/mL
Manhica, Mozambique (cohort 1) [[7],[14]]	1,589 (768)	RTS,S/AS02A	35 (24 to 48) months	40%	0,1,2 months	191 (9 to 916) EU/mL
Ilha Josina, Mozambique (cohort 2) [[7],[14]]	411 (196)	RTS,S/AS02A	36 (24 to 45) months	45%	0,1,2 months	266 (16 to 1,390) EU/mL
Kilifi, Kenya [[6],[15]]	447 (209)	RTS,S/AS01E	11 (8 to 14) months	35%	0,1,2 months	580 (104 to 1,922) EU/mL
Korogwe, Tanzania [[6]]	447 (224)	RTS,S/AS01E	12 (9 to 15) months	15%	0,1,2 months	493 (138 to 1,768) EU/mL
Kintampo, Ghana [[10]]	180 (180)	RTS,S/AS02D and RTS,S/AS01E	11 (8 to 14) months	80%	0,1,2 and 0,1,7 months	465 (73 to 2,632)^b^ EU/mL
Kumasi, Ghana [[10]]	270 (270)	RTS,S/AS02D and RTS,S/AS01E	11 (7 to 13) months	35%	0,1,2 and 0,1,7 months	460 (84 to 1,785)^b^ EU/mL
Lambaréné, Gabon [[9]]	180 (180)	RTS,S/AS02D and RTS,S/AS01E	38 (31 to 48) months	5%	0,1,2 months	198 (32 to 888) EU/mL
Bagamoyo, Tanzania [[8]]	209 (136)	RTS,S/AS01E	1.8 (1.7 to 1.9) months	30%	0,1,2 and 0,1,7 months^c^	167 (14 to 934)^b^ EU/mL
Lambaréné, Gabon [[8]]	215 (139)	RTS,S/AS01E	1.5 (1.4 to 1.7) months	5%	0,1,2 and 0,1,7 months^c^	337 (97 to 1,836)^b^EU/mL
Kintampo, Ghana [[8]]	81 (52)	RTS,S/AS01E	1.6 (1.5 to 1.8) months	80%	0,1,2 and 0,1,7 months^c^	70 (11 to 455)^b^ EU/mL
Mozambique infants [[16]]	214 (98)	RTS,S/AS02D	1.8 (1.8 to 2.1) months	45%	0,1,2 months	211 (6 to 1,008) EU/mL
Bagamoyo, Tanzania [[11]]	340 (157)	RTS,S/AS02D	1.9 (1.8 to 2) months	30%	0,1,2 months^c^	87 (1 to 572)^b^ EU/mL

For participants receiving at least one dose of RTS,S the peak anti-CSP antibody titre following vaccination is presented as the median and 95% range within the cohort at each trial site. ^a^Age-corrected parasite prevalence in 2- to 10-year olds taken from Malaria Atlas Project [[17]]; ^b^indicates peak anti-CSP antibody titre in the cohort vaccinated through a 0, 1, 2 month schedule; ^c^indicates co-administration with the EPI vaccines (diphtheria, tetanus, pertussis, hepatitis B, and *Haemophilus influenza* type b). CSP, circumsporozoite protein; EPI, expanded programme on immunization; EU, ELISA units; IQR, interquartile range.

### Immunogenicity

The method used for measuring anti-CSP antibodies was standardised and conducted in a single laboratory [[18]], except for samples from The Gambia which were analysed in the Walter Reed Army Institute of Research [[12]]. For each participant receiving at least two doses of RTS,S/AS01 or RTS,S/AS02 we took the anti-CSP antibody titre (CSP_peak_) measured within 21 to 30 days of the final dose to be the peak titre. Data from a fourth booster dose administered to some participants 14 months after the third dose were not included [[12]].

### Statistical methods

We examined the effects of the following covariates on CSP_peak_: adjuvant (AS01 *versus* AS02), age at vaccination, site-specific transmission intensity, dosing schedules (0, 1, 2 *versus* 0, 1, 7 months), number of doses received and co-administration of other vaccines. Participants were categorised according to age as follows: infants (≤3 months); children (>3 months and <5 years); and adults (>18 years). For each trial site, the age-corrected estimated parasite prevalence in 2- to 10-year olds in 2010 was obtained from the nearest location from the Malaria Atlas Project [[17]] as a proxy for transmission intensity. Trial site was included as a random effect to account for additional heterogeneity not captured by the fixed effects.

Following vaccination, the decay of antibody titres has been observed to have a short-lived phase (with titres decaying rapidly in the first few weeks), and a long-lived phase responsible for sustained vaccine-induced immunity, as has previously been observed for vaccine-induced responses to other infections [[19]]. To obtain estimates of anti-CSP antibody levels over time, we fitted a bi-phasic exponential decay model [[20]] to the anti-CSP antibody titres from all participants with at least two measurements. Following vaccination an individual’s antibody titre CSP(*t*) is assumed to decay from CSP_peak_ as follows:

(1) $$\mathrm{CSP}\left(t\right)=\mathrm{CS}P_{\mathrm{peak}}\left(\rho e^{-\log\left(2\right)\frac{t}{d_{s}}}+\left(1-\rho \right)e^{-\log\left(2\right)\frac{t}{d_{l}}}\right)$$

where *d*_*s*_ and *d*_*l*_ are the half-lives of the short-lived and long-lived components of the antibody response, and ρ is the proportion of the antibody response that is short-lived. Three studies included extended follow-up for longer than one year [[8],[14],[15]]. The model was fitted in a Bayesian framework using Markov Chain Monte Carlo (MCMC) methods with mixed effects used to capture between-individual variation [see Additional file 2].

We used the model-predicted anti-CSP antibody titres over time to estimate a dose–response curve for the relationship between antibody levels and protection from infection and disease using survival analysis methods [[21],[22]]. Vaccine efficacy against infection *V*(*t*) is estimated from antibody titre *CSP*(*t*) according to the following dose–response curve:

(2) $$V\left(t\right)=V_{\text{max}}\left(1-\frac{1}{1+{\left(\frac{\mathrm{CSP}\left(t\right)}{\beta }\right)}^{\alpha }}\right)$$

where α and β are shape parameters to be estimated, and *V*_max_ is the maximum efficacy against infection [[21]].

We assume that the entomological inoculation rate (EIR) varies between individuals to capture known heterogeneity in exposure to mosquito bites. Despite the reported seasonality in *P. falciparum* exposure in some of the trial sites, we make the simplifying assumption that EIR is constant over time. The rate at which an individual is exposed to malaria is then a function of (1) the EIR at the trial site and (2) their age (to account for age-dependency in biting rates). The probability that exposure results in infection is reduced by the dose–response function for vaccine efficacy in equation (2). The probability that an infection will progress to an episode of clinical malaria will be determined by a participant’s level of naturally-acquired immunity which is estimated using a previously published model [[5]]. Finally, the probability that a case of clinical malaria is observed is modified by a fixed effect for active or passive case detection. Parameters were estimated by fitting to the trial data in a Bayesian MCMC framework. Best fit parameters were taken to be the medians of the estimated posterior distributions. Parameters are presented with 95% credible intervals (CrI), the Bayesian analogue of confidence intervals (CI). Further details are in Additional file 2.

To assess the fit of the final model each of the phase 2 trials was re-simulated using the best fit parameters, and the results were compared to published vaccine efficacies. For each trial, the participants’ peak anti-CSP antibody titre was extracted and the incidence of infection and clinical malaria simulated. Data were simulated 1,000 times, each time recording the simulated vaccine efficacy [see Additional file 2].

### Estimates of efficacy and cases averted

Finally we used our fitted model to predict the expected pattern of vaccine efficacy decay against infection and clinical disease, and the cumulative number of cases averted in different transmission settings. The mean and variance of anti-CSP antibody titres following vaccination with RTS,S/AS01 by age (infants ≤3 months; children >3 months and <5 years) and co-administration (EPI vaccines or none) were used as an input (Table 2 and Additional file 2: Table S3). We used best fit parameters for the decay in antibody levels over time and the relationship between antibody levels and protection from infection and disease. Numbers of cases averted were estimated as the expected number of cases in an unvaccinated individual compared to an RTS,S vaccinated individual.

**Table 2:** Estimates of the impact of covariates on peak anti-CSP antibody titre following the final vaccine dose

**Covariate**	**Model 1 (N = 2,659)**		**Model 2 (N = 1,515)**	
	**Estimate (95% CI)**	***P*** **value**	**Estimate (95% CI)**	***P*** **value**
Children (>3 months and <5 years)	2.59 (2.27, 2.91)	<0.001	2.50 (2.12, 2.87)	<0.001
Infants (<3 months)	- 0.49(−0.96, −0.02)	0.04	- 0.41 (−0.91, 0.09)	0.11
Adults (>18 years)	- 1.33 (−1.95, −0.69)	0.002	- 1.18 (−1.87, −0.48)	0.01
log_10_(CSP_base_)*children	0.05 (−0.04, 0.14)	0.28	- 0.03 (−0.14, 0.09)	0.66
log_10_(CSP_base_)*infants	- 0.58 (−0.76, −0.40)	<0.001	- 0.48 (−0.69, −0.27)	<0.001
log_10_(CSP_base_)*adults	0.24 (0.07, 0.41)	0.006	0.30 (0.11, 0.48)	0.002
Adjuvant (AS02 *versus* AS01)	- 0.13 (−0.20, −0.05)	<0.001	- 0.12 (−0.19, −0.05)	<0.001
Parasite prevalence	0.30 (−0.29, 0.89)	0.32	0.30 (−0.32, 0.93)	0.35
Doses (2 *versus* 3)	- 0.46 (−0.57, −0.36)	<0.001	- 0.47 (−0.57, −0.37)	<0.001
Schedule (017 m *versus* 012 m)	- 0.85 (−0.93, −0.77)	<0.001	- 0.84 (−0.92, −0.76)	<0.001
Co-administration	- 0.50 (−1.04, 0.03)	0.095	- 0.52 (−1.12, 0.07)	0.13
log_10_(HBs_base_)*children	–	–	0.05 (0.02, 0.09)	0.005
log_10_(HBs_base_)*infants	–	–	- 0.04 (−0.14, 0.05)	0.34
log_10_(HBs_base_)*adults	–	–	- 0.12 (−0.20, −0.04)	0.002

The RTS,S vaccine effect is the estimated peak anti-CSP antibody titre for a child receiving three doses of RTS,S/AS01 administered via a 0, 1, 2 month schedule without co-administration of other vaccines. The estimates presented are the regression coefficients for log_10_(CSP_peak_/(EU/mL)) against the corresponding covariates. The pre-vaccination anti-CSP and anti-HBs antibody titres are denoted CSP_base_ and HBs_base_, respectively. Model 1 was fitted to all phase 2 trial participants with measurements of CSP_peak_ and CSP_base_. Model 2 extends Model 1 by investigating the dependence of peak anti-CSP antibody titre on pre-vaccination anti-HBs titre, and includes all trial participants with measurements of CSP_peak_, CSP_base_ and HBs_base_. For categorical variables regression coefficients describe the association between the relevant covariate and log_10_(CSP_peak_). The regression coefficient for parasite prevalence indicates that an increase in prevalence from 0 to 1 is associated with an increase in log_10_(CSP_peak_) of 0.30. CI, confidence interval; CSP, circumsporozoite protein; HBs, hepatitis B surface antibody.

## Results

### Immunogenicity of the vaccine

Table 2 shows the impact of the tested covariates on the peak anti-CSP antibody titre following the final vaccine dose. Immunogenicity was highest among children: geometric mean anti-CSP antibody titre 465 (95% range: 41 to 5,305) EU/mL, intermediate for infants: 333 (95% range: 29 to 3,847) EU/mL and lowest for adults: 42 (95% range: 4 to 484) EU/mL. The adjuvant AS01 was significantly more immunogenic than AS02, three doses were more immunogenic than two, and RTS,S was more immunogenic when 0, 1, 2 month schedules were used rather than 0, 1, 7 month schedules. Co-administration of RTS,S with other vaccines reduced immunogenicity in infants: 82 (95% range: 7 to 941) EU/mL versus 333 (95% range: 29 to 3,847) EU/mL (*P* = 0.095, Table 2) although this was not statistically significant.

Higher pre-vaccination anti-CSP titres were associated with lower peak anti-CSP titres in infants (*P* <0.001), but with higher peak anti-CSP titres in children (not significant, *P* = 0.28) and adults (*P* = 0.006). Higher pre-vaccination anti-hepatitis B surface antibody (HBs) titres due to prior hepatitis B vaccination were associated with higher peak anti-CSP titres in children (*P* = 0.005) and lower peak anti-CSP titres in adults (*P* = 0.002). There was no statistically significant association between peak anti-CSP titres and past exposure as measured by estimated parasite prevalence [[17]] (*P* = 0.32). Although baseline anti-CSP antibody titres are imperfect markers of past *P. falciparum* exposure, they do suggest higher levels of immunogenicity in previously exposed adults (Table 2).

### Decay in vaccine-induced antibody titres

**Figure 1:**
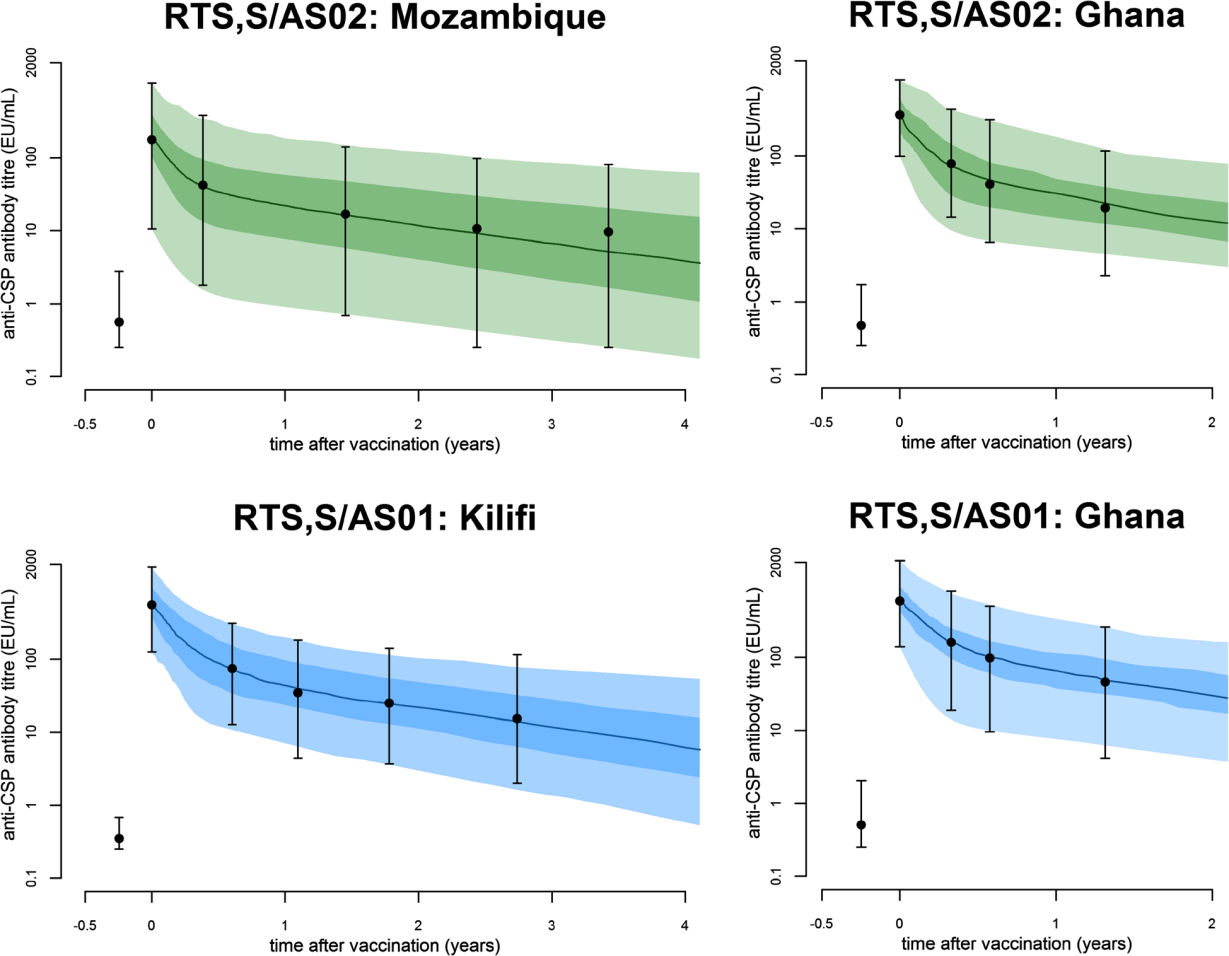
**Anti-CSP antibody titre dynamics.** Anti-CSP antibody titre dynamics for trials with extended follow-up for longer than one year in Mozambique [[14]], Kilifi [[23]] and Ghana [[10]]. The black bars denote the median and 95% ranges from the data. The green and blue curves denote the antibody titres predicted by equation (1) for RTS,S/AS02 and RTS,S/AS01, respectively. Dark and light shading represents 50% and 95% of the model-predicted variation in antibody titres. We estimated that 82% (95% CrI: 80% to 83%) of the RTS,S induced antibody response was short-lived with half-life 46 (95% CrI: 43 to 49) days, the rest being long-lived with half-life 594 (95% CrI: 551 to 645) days. CrI, credible interval; CSP, circumsporozoite protein.

A bi-phasic exponential model for antibody decay stratified by adjuvant system was fitted to the data [see Additional file 2: Table S6]. Figure 1 shows the model fit to the data from the three studies with extended follow-up. The RTS,S/AS02 induced anti-CSP antibodies showed a lower peak following vaccination than the RTS,S/AS01 induced antibodies (Table 2), but the pattern of antibody decay leads to similar antibody titres three years after vaccination. The bi-phasic pattern of decay in vaccine-induced antibody titres is qualitatively similar to the decay of naturally-acquired antibody responses in African children [[20]].

### Association between antibodies and protection from infection

**Figure 2:**
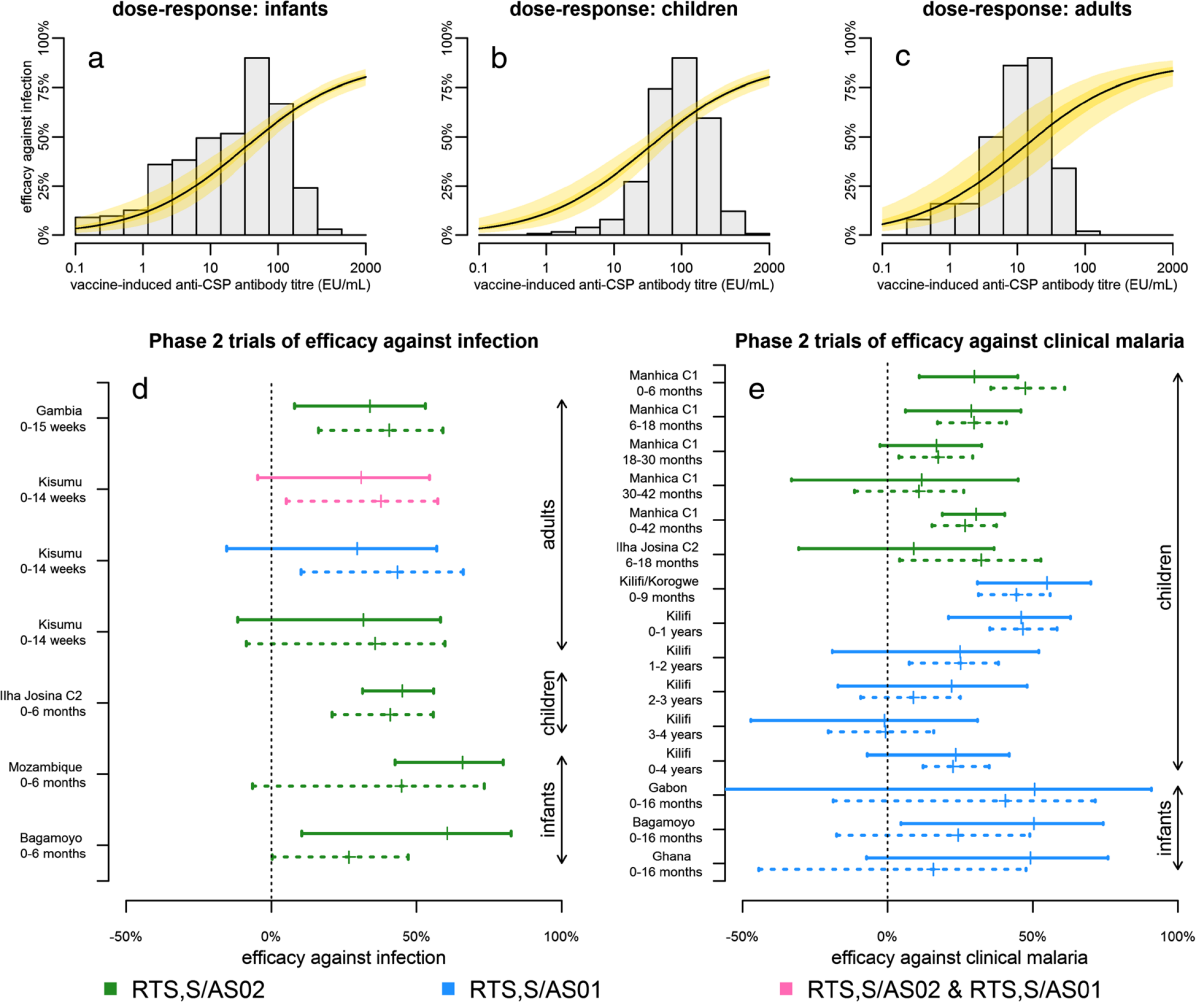
**Association between anti-CSP antibody titre and protection. (a,b,c)** Estimated dose–response curves from equation (2) for the association between anti-CSP antibody titre CSP and vaccine efficacy against infection *V* (the probability that an infection is prevented by RTS,S induced responses) for infants (<3 months), children (>3 months and <5 years), and adults (>18 years). The yellow shaded region denotes the 95% credible interval. The grey histograms denote the observed distribution of each trial participant’s average anti-CSP titre during the first year of follow-up. **(d)** Comparison of observed (solid lines) and simulated (dashed lines) efficacy against infection from phase 2 trials. **(e)** Comparison of observed (solid lines) and simulated (dashed lines) efficacy against clinical malaria. The range of the solid lines represents the 95% confidence intervals of vaccine efficacy observed in phase 2 trials. The dashed lines represent the 95% range due to stochastic variation in simulated vaccine efficacy in cohorts of equal size to the original trial using the model with posterior median parameter estimates. CSP, circumsporozoite protein.

Figure 2a-c shows the estimated dose–response relationships between anti-CSP antibody titre and protection for infants, children and adults. An anti-CSP antibody titre of 51 (95% CrI: 29 to 85) EU/mL was estimated to prevent 50% of infections in children and infants, and an anti-CSP antibody titre of 19 (95% CrI: 4 to 83) EU/mL was estimated to prevent 50% of infections in adults [see Additional file 2: Table S8]. Vaccine efficacy against infection and clinical malaria predicted by simulation using the estimated dose–response relationships were compared with observed efficacies in phase 2 trials (Figure 2d-e). Notably, the model captures the decline in efficacy against clinical malaria over time in a cohort of children in Kilifi, Kenya [[15]]. The pattern of declining efficacy in Manhica cohort 1, Mozambique [[14]] is also replicated, although efficacy in the first six months of follow-up is overestimated. This is possibly due to a reduction in transmission intensity in Manhica due to increased bed net coverage and other interventions not accounted for in the model [[24]].

### Dependence of vaccine efficacy on age, exposure and time since vaccination

**Figure 3:**
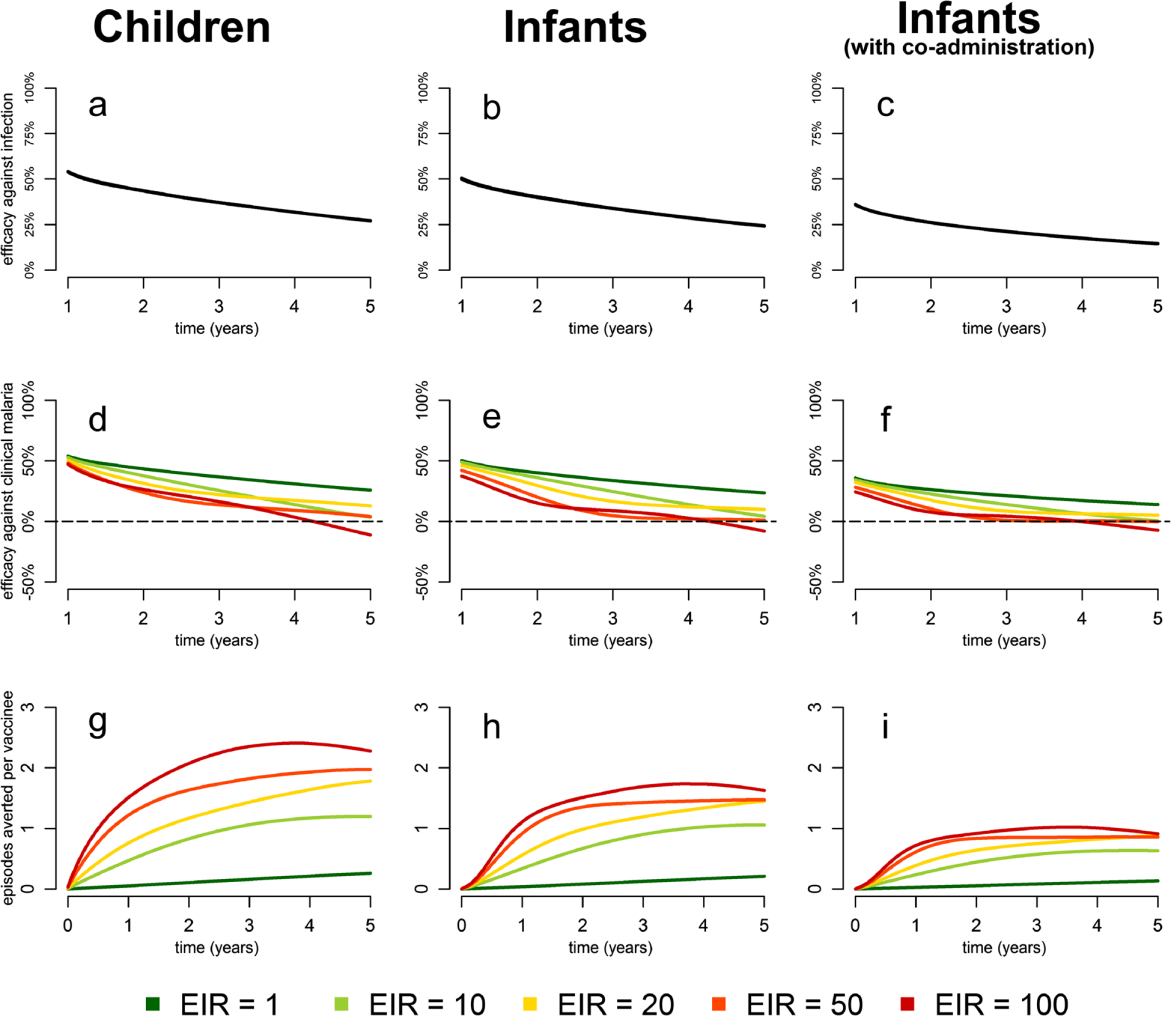
**Predicted vaccine efficacy and cumulative cases averted. (a,b,c)** Model predicted change in vaccine efficacy against infection over time for children (>3 months and <5 years), infants (≤3 months) and infants co-administered with EPI vaccines. Vaccine efficacy against infection at year *x* is the vaccine efficacy against all infections during the period (*x*-1,*x*) years. **(d,e,f)** Model predicted change in vaccine efficacy against episodes of clinical malaria over time for children, infants, and infants co-administered with EPI vaccines. Vaccine efficacy against clinical malaria at year *x* is the efficacy against all episodes during the period (*x*-1,*x*) years. **(g,h,i)** Cumulative episodes of clinical malaria averted by RTS,S per fully vaccinated child or infant. Cases averted at year *x* is the expected difference in the number of clinical episodes in the period (0,*x*) years between a vaccinated and unvaccinated child or infant. Cases are assumed to be detected via weekly active case detection. The uncertainty associated with these estimates is presented in Additional file 2: Figure S5. EPI, expanded programme on immunization.

Figure 3a-c shows the model predicted decline in efficacy against infection over time. Efficacy decreases over time due to the decay of RTS,S induced anti-CSP antibodies (vaccine waning), with efficacy against infection in children dropping from 54% (95% CrI: 48% to 59%) in the first year of follow-up to 27% (95% CrI: 20% to 34%) in the fifth year of follow-up. Figure 3d-f shows the predicted change in efficacy against clinical malaria over time for a range of transmission intensities. There is substantial variation in the predicted patterns of waning at different transmission intensities due to the model-predicted differential acquisition of immunity between the vaccine and control cohorts. Those in the control cohort are exposed to more infections and, hence, are predicted to develop a higher level of natural immunity over time. At EIR = 1 infectious bite per year (ibpy), the pattern of decay of efficacy against clinical malaria closely tracks the pattern observed for efficacy against infection as there is very little acquisition of natural immunity. At moderate transmission intensities (EIR = 10 to 20 ibpy), our model predicts that efficacy against clinical malaria will decay to near zero over four years. At high transmission intensities (EIR ≥50 ibpy), efficacy against clinical malaria decays to near zero after approximately three years, after which we predict that the vaccine cohort will experience more episodes of clinical malaria than the control cohort. Figure 3g-i shows the estimated number of cumulative episodes of clinical malaria averted over time. We estimate that over a five-year follow-up period in a site with EIR = 20 ibpy and weekly active case detection, RTS,S averts 1,782 (95% CrI: 1,408 to 2,089) cases per 1,000 vaccinated children, 1,452 (95% CrI: 1,096 to 1,767) cases per 1,000 vaccinated infants, and 887 (95% CrI: 604 to 1,218) cases per 1,000 infants co-administered with EPI vaccines. At high transmission intensities (>20 ibpy), differences in the rate of acquisition of immunity can lead to higher incidence in the vaccine group compared to the control group at longer term, although the cumulative number of cases averted remains positive.

## Discussion and conclusions

Our results demonstrate that variations in vaccine efficacy can be explained by the magnitude and duration of the RTS,S-induced anti-CSP antibody response. In turn, immunogenicity depends on age, pre-existing immunity, co-administration of other vaccinations, dosing regimen and adjuvant system. Children aged three months to five years had significantly higher vaccine-induced anti-CSP antibody titres than infants younger than three months, who in turn had higher vaccine-induced anti-CSP titres than adults. Also, the associations between pre-vaccination anti-CSP antibody titres and peak titres were negative in infants, positive in older children and non-significant in adults. Infants are thought to have less well developed capacity for immunological responses than older children [[25]], and our data also suggest that immunogenicity in infants is reduced further where maternal exposure to malaria was high. This might be attributable to passive immunity inhibiting the development of new immunological responses, as has been described for hepatitis B vaccination [[26]]. In contrast, the positive association between pre-vaccination anti-CSP titres and peak anti-CSP titres in children and adults suggests improved immunogenicity in subjects with pre-existing naturally-acquired immunity [[3]]. A positive association was also observed between pre-vaccination anti-HBs titres and peak anti-CSP titres in children, a result previously reported in a cohort of Gabonese children [[9]]. Hypothetical mechanisms underlying this association include: (1) HBs-primed B cells expressing anti-HBs antibodies capturing the RTS,S antigen and ensuring efficient presentation for CSP-specific T-cell priming; and (2) HBs-specific CD4 memory T-cells providing more rapid T-cell help to CSP-specific B cells upon stimulation by the HBs antigen in RTS,S [[9]].

Linear regression models without a random effect for trial site predict a statistically significant association between peak anti-CSP antibody titre and co-administration status (76% reduction, *P* <0.001, Additional file 2: Table S2). However, this effect may be attributable to inter-trial site variation and was not statistically significant when random effects by trial site were incorporated in the regression models (68% reduction, *P* = 0.095, Additional file 2: Table S1). The cohort of infants receiving RTS,S/AS02D co-administered with EPI vaccines in Tanzania [[11]] had substantially lower immunogenicity than a comparable cohort of infants vaccinated with RTS,S/AS02D without co-administration in Mozambique [[16]]: 87 (95% range: 1 to 572) EU/mL *versus* 211 (95% range: 6 to 1,008) EU/mL, respectively. Statistically significant reductions in immunogenicity due to co-administration have been observed in vaccine trials of infants receiving pneumococcal [[27]] and *Haemophilus influenza* type b [[28]] vaccines. Hence, in context, an impact of co-administration is possible despite the lack of statistical significance.

We found anti-CSP antibody titres to be associated with protection against infection and episodes of clinical malaria [[29],[30]], with the estimated dose–response relationship predicting increasing efficacy with increasing antibody titre across multiple trial sites (Figure 2). In particular, three of the four Prentice criteria [[30]] are satisfied: (1) RTS,S vaccination affects outcome (infection or clinical malaria); (2) RTS,S affects surrogates (anti-CSP antibodies); and (3) surrogates affect outcome (anti-CSP antibodies are associated with protection). The fourth criterion, that conditional upon the surrogates outcomes are independent of vaccination status, is less easily satisfied by the diverse data from the phase 2 trials. This equates to whether anti-CSP antibody titres can be used to predict vaccine efficacy. The comparison between observed and model predicted vaccine efficacies in Figure 2d,e suggests the criterion is satisfied, although there is likely to be an important role for CSP-specific CD4^+^ T cell responses which has not been accounted for. The association between anti-CSP antibody titres and protection from infection is consistent with data from other vaccine studies [[31],[32]], as well as data from mouse models where RTS,S-induced anti-CSP antibodies have been shown to inhibit sporozoite invasion [[33]]. There was no evidence for a threshold anti-CSP antibody titre above which large increases in efficacy are achieved [[34]]. The smooth, albeit non-linear, shape of the dose–response curve is consistent with RTS,S having the profile of a leaky vaccine, but with substantial variation in efficacy.

The use of anti-CSP antibodies as the sole marker of the RTS,S-induced immune response constitutes a potential limitation of this analysis, as RTS,S-induced CSP-specific CD4^+^ T cells have been shown to be associated with protection from *P. falciparum* infection and episodes of clinical malaria [[35]]. However, analysis of immunological data from a challenge trial of RTS,S [[36]] suggests that anti-CSP antibody titres play a dominant role in protection from infection [[21]]. Furthermore, the magnitude of the RTS,S induced antibody and cell-mediated responses are correlated [[36]], so anti-CSP antibody titres may act as markers for cellular immunity as well as antibody-mediated immunity. Efficacy against clinical malaria has also been shown to correlate with peripheral blood monocyte-to-lymphocyte ratios before vaccination [[37]]. Total anti-CSP immunoglobulin G (IgG) responses were measured which poses another limitation as the duration and effectiveness of the RTS,S-induced antibody responses may depend on IgG subclass [[38],[39]].

By combining our model for the decay of anti-CSP antibody titres, the estimated relationship between antibodies and efficacy, and a model for the acquisition of immunity to clinical malaria [[5]], we were able to demonstrate associations between the magnitude and duration of efficacy on a number of covariates. Most striking is the dependence of efficacy against clinical malaria on estimated transmission intensity and EPI vaccine co-administration status. Increased exposure resulted in lower initial efficacy against clinical malaria in the first year of follow-up, as well as a shorter duration of protection. At longer term, the combination of decaying anti-CSP antibody titres in the vaccinated cohort and increased naturally-acquired immunity in the control cohort resulted in waning of the efficacy of RTS,S to zero or below, over a duration of follow-up dependent on transmission intensity (Figure 3). The prediction of higher incidence of clinical malaria in the vaccine cohort compared to the control cohort during extended follow up in high transmission settings is consistent with observations of the incidence of malaria in a high exposure cohort in Kenya [[15]].

The variation in the characteristics of the phase 2 trials poses a potential limitation to this analysis. Differences in factors such as study populations, malaria transmission intensity, adjuvant formulation and co-administration of other vaccines complicate the analysis. For example, data on adults vaccinated with RTS,S/AS02 and followed for infection [[13]] are combined with data on infants vaccinated with RTS,S/AS01 and followed for clinical malaria [[8]]. Despite these difficulties, there is an advantage to such diversity of data as it allows systematic comparison between participants receiving different vaccination regimens at varying ages. This is in contrast to data from ongoing phase 3 trials which have more standardised vaccination regimens in more homogeneous populations [[1],[2]]. Comparison of the varied participants in phase 2 trials with participants in phase 3 trials may provide explanations for the lower immunogenicity and efficacy observed in infants compared to children, suggesting roles for the co-administration of EPI vaccines and reductions in the immunogenicity of RTS,S-induced responses due to interference by maternally-acquired antibodies. Knowledge of the varied determinants of vaccine efficacy will allow identification of sub-populations in which RTS,S will be most effective or cost-effective [[40]]. Finally, the results presented in this analysis will need to be confirmed against individual level data from participants in the ongoing phase 3 trials once this becomes available [[1],[2]].

## Competing interests

The analysis for this study was conducted following a call for proposals initiated and facilitated by GSK Vaccines. Employees of GSK Vaccines reviewed and commented on early draft manuscripts, but were not involved in the final approval of the manuscript.

## Authors’ contributions

MTW, PB and ACG designed the study. MTW performed the analysis. JTG and JJA advised on statistical analysis. PB, AO, KP, JL, NS, SA, NO, STA, BL, KPA, SOA, EM, TA, DA, JS and JJA collected the data. MTW, PB and ACG prepared the manuscript. All authors read and approved the final manuscript.

## Additional files

## Supplementary Material

Additional file 1 Information on the ethical approval regarding the trials including in this analysis. Click here for file

Additional file 2 Supplementary statistical methods and additional analyses. Click here for file
