# Supplementary material for: A combined analysis of immunogenicity, antibody kinetics and vaccine efficacy from phase 2 trials of the RTS,S malaria vaccine
Source: BMC Med. 2014 Jul 10;12:117. doi: 10.1186/s12916-014-0117-2 (PMC4227280; doi:10.1186/s12916-014-0117-2)
Supplement: Additional file 1 — Information on the ethical approval regarding the trials including in this analysis. [file s12916-014-0117-2-S1.docx]

**A combined analysis of immunogenicity, antibody kinetics and vaccine efficacy from phase 2 trials of the RTS,S malaria vaccine**

**Additional file 1:** Information on the ethical approval regarding the trials including in this analysis

The manuscript contains data from a number of different phase 2 clinical trials of the RTS,S malaria vaccine. Each of the individual trials obtained approval from at least one institutional review board. Details are provide below.

1. Bejon P, Lusingu J, Olotu A, Leach A, Lievens M, Vekemans J, Mshamu S, Lang T, Gould J, Dubois M, Demoitié MA, Stallaert JF, Vansadia P, Carter T, Njuguna P, Awuondo KO, Malabeja A, Abdul O, Gesase S, Mturi N, Drakeley CJ, Savarese B, Villafana T, Ballou WR, Cohen J, Riley EM, Lemnge MM, Marsh K, von Seidlein L: **Efficacy of RTS,S/AS01E vaccine against malaria in children 5 to 17 months of age.** *N Engl J Med* 2008, 359:2521–2532.

   Approval was obtained from the Kenyan Medical Research Institute National Ethics Committee, the Tanzanian Medical Research Coordinating Committee, the Central Oxford Research Ethics Committee, the London School of Hygiene and Tropical Medicine Ethics Committee, and the Western Institutional Review Board in Seattle. An independent data and safety monitoring board and local safety monitors were appointed. The study was conducted in accordance with the Helsinki Declaration of 1964 (revised in 1996) and according to Good Clinical Practice guidelines.
2. Alonso PL, Sacarlal J, Aponte JJ, Leach A, Macete E, Milman J, Mandomando I, Spiessens B, Guinovart C, Espasa M, Bassat Q, Aide P, Ofori-Anyinam O, Navia MM, Corachan S, Ceuppens M, Dubois MC, Demoitié MA, Dubovsky F, Menéndez C, Tornieporth N, Ballou WR, Thompson R, Cohen J: **Efficacy of the RTS,S/AS02A vaccine against Plasmodium falciparum infection and disease in young African children: randomised controlled trial.** *Lancet* 2004, 364:1411–1420.

   The protocol was approved by the national Mozambican ethics review committee, the Hospital Clinic of Barcelona ethics review committee, and the PATH human subjects protection committee. The trial was undertaken according to the International Conference on Harmonisation Good Clinical Practice guidelines and was monitored by GSK Biologicals. A local safety monitor and a data and safety monitoring board closely reviewed the conduct and results of the trial.
3. Asante KP, Abdulla S, Agnandji S, Lyimo J, Vekemans J, Soulanoudjingar S, Owusu R, Shomari M, Leach A, Jongert E, Salim N, Fernandes JF, Dosoo D, Chikawe M, Issifou S, Osei-Kwakye K, Lievens M, Paricek M, Möller T, Apanga S, Mwangoka G, Dubois MC, Madi T, Kwara E, Minja R, Hounkpatin AB, Boahen O, Kayan K, Adjei G, Chandramohan D, et al: **Safety and efficacy of the RTS,S/AS01(E) candidate malaria vaccine given with expanded-programme-on-immunisation vaccines: 19 month follow-up of a randomised, open-label, phase 2 trial.** *Lancet Infect Dis* 2011, 11:741–749.

   The trial was done in accordance with the Helsinki Declaration of 1964 (revised in 1996)18 and according to Good Clinical Practice guidelines.19 Approval was obtained from the local and national ethics committees relevant to each site, the London School of Hygiene and Tropical Medicine Ethics Committee, UK, the Swiss Tropical Institute Committee, Switzerland, and The Western Institutional Review Board, USA. The design, conduct, and results of the trial were overseen by a formally constituted independent data monitoring committee. The study was done under US Food and Drug Administration and national regulatory oversight as per existing regulations. The Ghana Food and Drugs Board, the National Institute for Medical Research of Tanzania, and the Ministry of Health in Gabon reviewed and approved the study before it started.
4. Lell B, Agnandji S, von Glasenapp I, Haertle S, Oyakhiromen S, Issifou S, Vekemans J, Leach A, Lievens M, Dubois MC, Demoitie MA, Carter T, Villafana T, Ballou WR, Cohen J, Kremsner PG**: A randomized trial assessing the safety and immunogenicity of AS01 and AS02 adjuvanted RTS,S malaria vaccine candidates in children in Gabon.** *Plos One* 2009, 4:e7611.

   The study protocol was approved by the ethics committee of the International Foundation of the Albert Schweitzer Hospital of Lambaréné and the Western Institutional Review Board, USA. The trial was undertaken according to the International Conference on Harmonisation of Good Clinical Practice guidelines and was monitored by GSK Biologicals, Rixensart, Belgium. A local safety monitor and a data and safety monitoring board closely reviewed the conduct and results of the trial.
5. Owusu-Agyei S, Ansong D, Asante K, Owusu SK, Owusu R, Brobby NAW, Dosoo D, Akoto AO, Osei-Kwakye K, Adjei EA, Boahen KO, Sylverken J, Adjei G, Sambian D, Apanga S, Kayan K, Vekemans J, Ofori-Anyinam O, Leach A, Lievens M, Demoitie MA, Dubois MC, Cohen J, Ballou WR, Savarese B, Chandramohan D, Gyapong JO, Milligan P, Antwi S, Agbenyega T, et al: **Randomized controlled trial of RTS,S/AS02(D) and RTS,S/AS01(E) malaria candidate vaccines given according to different schedules in Ghanaian children.** *Plos One* 2009, 4:7302.

   The protocol was approved by the Food and Drugs Board, Ghana; the Ghana Health Service Ethical Review Committee, Accra, Ghana; the KHRC Institutional Ethics Committee, the KHRC Scientific Review Committee; the Committee on Human Research Protection and Ethics, SMS, KNUST, Kumasi, Ghana; the London School of Hygiene and Tropical Medicine (LSHTM) ethics committee, London, UK and the Western Institutional Review Board, Washington, USA. The trial was undertaken according to the International Conference on Harmonization, Good Clinical Practice guidelines and was monitored by GlaxoSmithKline (GSK) Biologicals. The study was overseen by a formally constituted Data Safety Monitoring Board (DSMB) operating under a charter. The DSMB reviewed safety data from a RTS,S/AS trial in older children [8] prior to authorising the start of this study, and from a subset of children post dose 1 and post dose 2, prior to progression to the next vaccination dose within this trial. A Local Safety Monitor was designated at each site whose overall role was to support the clinical investigator and to act as a link between the investigator and the DSMB.
6. Abdulla S, Oberholzer R, Juma O, Kubhoja S, Machera F, Membi C, Omari S, Urassa A, Mshinda H, Jumanne A, Salim N, Shomari M, Aebi T, Schellenberg DM, Carter T, Villafana T, Demoitié MA, Dubois MC, Leach A, Lievens M, Vekemans J, Cohen J, Ballou WR, Tanner M: **Safety and immunogenicity of RTS,S/AS02D malaria vaccine in infants.** *N Engl J Med* 2008, 359:2533–2544.

   The protocol was approved by the Ifakara Health Institute, the Western Institutional Review Board in the United States, the National Institute of Medical Research in Tanzania, the Institutional Review Board of the London School of Hygiene and Tropical Medicine, and the Swiss Tropical Institute through the local government ethics committee in Basel, Switzerland. The trial was undertaken in accordance with the provisions of the International Conference on Harmonisation and Good Clinical Practice guidelines and was monitored by the sponsor, GlaxoSmithKline Biologicals, which provided both the RTS,S/AS02D vaccine and the hepatitis B vaccine.
7. Bojang KA, Milligan PJ, Pinder M, Vigneron L, Alloueche A, Kester KE, Ballou WR, Conway DJ, Reece WH, Gothard P, Yamuah L, Delchambre M, Voss G, Greenwood BM, Hill A, McAdam KP, Tornieporth N, Cohen JD, Doherty T, RTS, S Malaria Vaccine Trial Team: **Efficacy of RTS,S/ASO2 malaria vaccine against Plasmodium falciparum infection in semi-immune adult men in The Gambia: a randomised trial.** *Lancet* 2001, 358:1927–1934.

   The study was approved by the Joint Gambia Government/MRC Ethics Committee, an independent data safety monitoring committee, and the institutional review boards of collaborating partners. The trial was done according to International Conference on Harmonisation Good Clinical Practice guidelines, and was monitored by WHO and GlaxoSmithKline Biologicals.
8. Polhemus ME, Remich SA, Ogutu BR, Waitumbi JN, Otieno L, Apollo S, Cummings JF, Kester KE, Ockenhouse CF, Stewart A, Ofori-Anyinam O, Ramboer I, Cahill CP, Lievens M, Dubois MC, Demoitie MA, Leach A, Cohen J, Ballou WR, Heppner DG Jr: **Evaluation of RTS,S/AS02A and RTS,S/AS01B in adults in a high malaria transmission area.** *PLoS One* 2009, 4:e6465.

   The protocol was approved by the KEMRI and Kenya National Ethical Review Committee, Nairobi, and the US Army Medical Research and Materiel Command’s Human Subjects Research Review Board, Fort Detrick, Maryland. The trial was undertaken according to the International Conference on Harmonization, Good Clinical Practice guidelines and was monitored by GSK Biologicals. A Local Safety Monitor and a Safety Monitoring Group closely reviewed the conduct and results of the trial.
9. Aponte JJ, Aide P, Renom M, Mandomando I, Bassat Q, Sacarlal J, Manaca MN, Lafuente S, Barbosa A, Leach A, Lievens M, Vekemans J, Sigauque B, Dubois MC, Demoitié MA, Sillman M, Savarese B, McNeil JG, Macete E, Ballou WR, Cohen J, Alonso PL: **Safety of the RTS,S/AS02D candidate malaria vaccine in infants living in a highly endemic area of Mozambique: a double blind randomised controlled phase I/IIb trial.** *Lancet* 2007, 370:1543–1551.

   The protocol (NCT00197028, BB-IND 10514) was approved by the Mozambican National Bioethics Committee, the Hospital Clinic of Barcelona Ethics Review Committee, and the PATH Human Subjects Protection Committee, and was implemented according to the International Conference of Harmonization Good Clinical Practices guidelines. GlaxoSmithKline (GSK) monitored the study. A local Safety Monitor and a Data and Safety Monitoring Board oversaw the design, conduct, and results of the trial.
