# Supplementary material for: A combined analysis of immunogenicity, antibody kinetics and vaccine efficacy from phase 2 trials of the RTS,S malaria vaccine
Source: BMC Med. 2014 Jul 10;12:117. doi: 10.1186/s12916-014-0117-2 (PMC4227280; doi:10.1186/s12916-014-0117-2)
Supplement: Additional file 2 — Supplementary statistical methods and additional analyses. [file s12916-014-0117-2-S2.docx]

**A combined analysis of immunogenicity, antibody kinetics and vaccine efficacy from phase 2 trials of the RTS,S malaria vaccine**

**Appendix: Extended statistical methods**

Michael T. White^1^*, Philip Bejon^2,3^, Ally Olotu^2^, Jamie T. Griffin^1^, Kalifa Bojang^4^, John Lusingu^5^, Nahya Salim^6^, Salim Abdulla^6^, Nekoye Otsyula^7^, Selidji T. Agnandji^8,9^, Bertrand Lell^8,9^, Kwaku Poku Asante^10^, Seth Owusu-Agyei^10^, Emmanuel Mahama^10^, Tsiri Agbenyega^11^, Daniel Ansong^11^, Jahit Sacarlal^12,13^, John J. Aponte^12,14^, Azra C. Ghani^1^

^1^MRC Centre for Outbreak Analysis and Modelling, Imperial College London, London, UK

^2^KEMRI-Wellcome Trust Research Programme, Kenya Medical Research Institute, Kilifi, Kenya

^3^Centre for Clinical Vaccinology and Tropical Medicine, University of Oxford, UK

^4^Medical Research Council Unit, Fajara, The Gambia

^5^National Institute for Medical Research, Tanga Centre, Tanga, Tanzania

^6^Ifakara Health Institute, Bagamoyo, Tanzania

^7^Kenya Medical Research Institute, and US Army Medical Research Unit–Kenya, Nairobi, Kenya

^8^Medical Research Unit, Albert Schweitzer Hospital, Lambaréné, Gabon

^9^Institute of Tropical Medicine, University of Tübingen, Tübingen, Germany

^10^Kintampo Health Research Centre, Kintampo, Ghana

^11^School of Medical Sciences, Kumasi, Ghana

^12^Centro de Investigação em Saúde de Manhiça, Manhiça, Mozambique

^13^Faculdade de Medicina, Universidade Eduardo Mondlane, Mozambique

^14^Barcelona Centre for International Health Research (CRESIB), Universitat de Barcelona, Barcelona, Spain

* Corresponding author:

Dr Michael T. White

MRC Centre for Outbreak Analysis and Modelling,

Department of Infectious Disease Epidemiology,

Imperial College London,

London, W2 1PG,

UK

e-mail: [m.white08@imperial.ac.uk](mailto:m.white08@imperial.ac.uk)

1. **Linear regressions for peak anti-CSP antibody titres**

Peak anti-CSP antibody titre following RTS,S vaccination (CSP_peak_) depends on a number of covariates, including age at vaccination and baseline anti-CSP and anti-HBs antibody titres prior to vaccination. Pre-vaccination anti-CSP and anti-HBs antibody titres are denoted CSP_base_ and HBs_base_, respectively. Figure S1 shows the dependence of the increase in anti-CSP antibody titre following vaccination on age category, CSP_base_, and HBs_base_. Figure S2 shows the effect of anti-CSP and anti-HBs antibody sero-positivity on anti-CSP antibody titres following RTS,S vaccination. Individuals are defined as anti-CSP antibody sero-negative if they had pre-vaccination anti-CSP antibody titre ≤ 0.25 EU/mL. Individuals are defined as anti-HBs antibody sero-negative if they had pre-vaccination anti-HBs antibody titre ≤ 5 EU/mL.

**
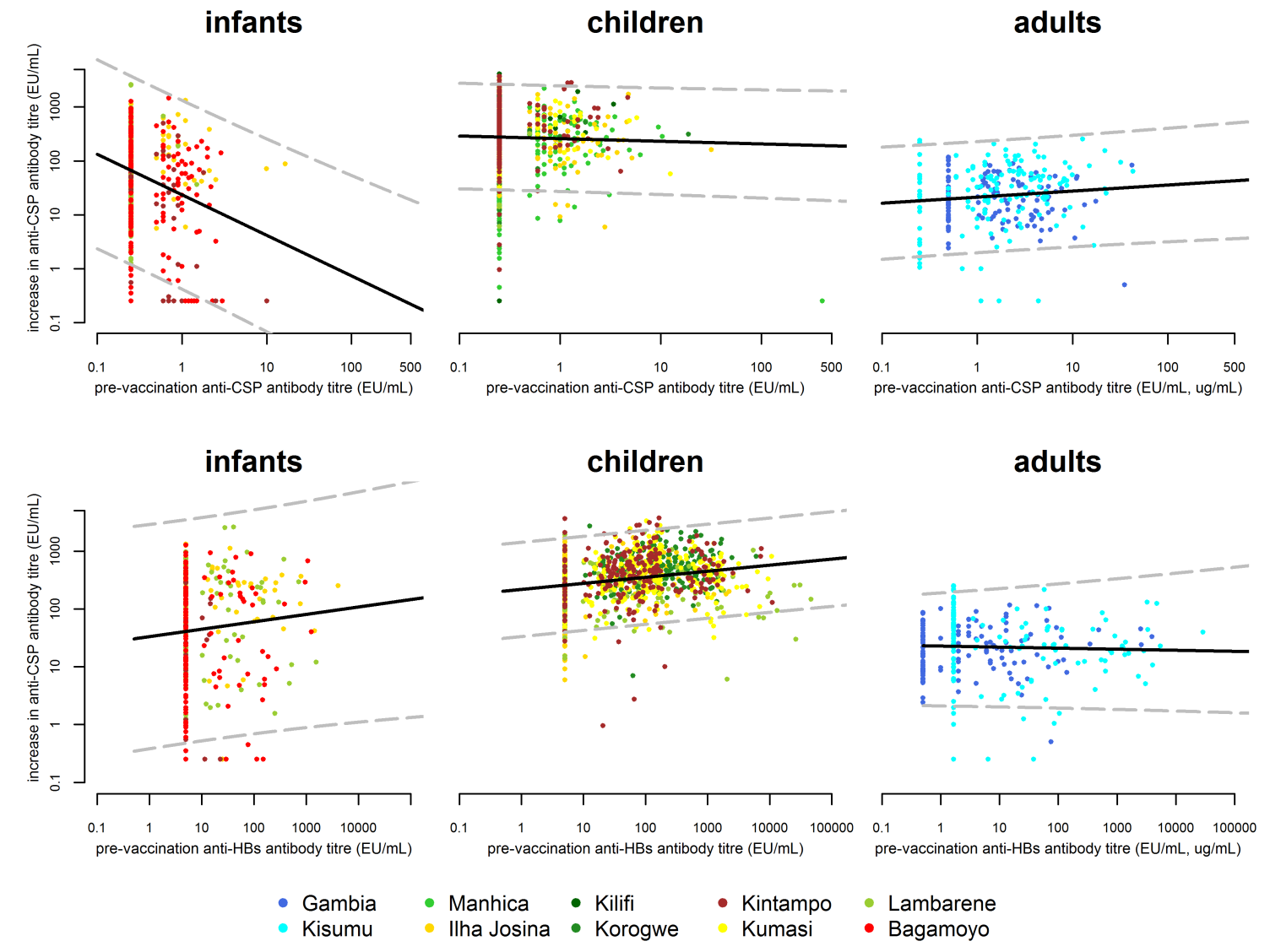
**

Figure S1: Association between increase in anti-CSP antibody titre following RTS,S vaccination and pre-vaccination anti-CSP and anti-HBs antibody titres stratified by age group. The lowest detectable anti-CSP antibody titre was 0.25 EU/mL except in the Gambian cohort. In the cohort of Gambian adults, antibody titres were measured in µg/mL, and the lower limit of detection was 0.5 µg/mL. Measurements of antibody titres in units of µg/mL and EU/mL are not necessarily equivalent but are plotted on the same scale.

**
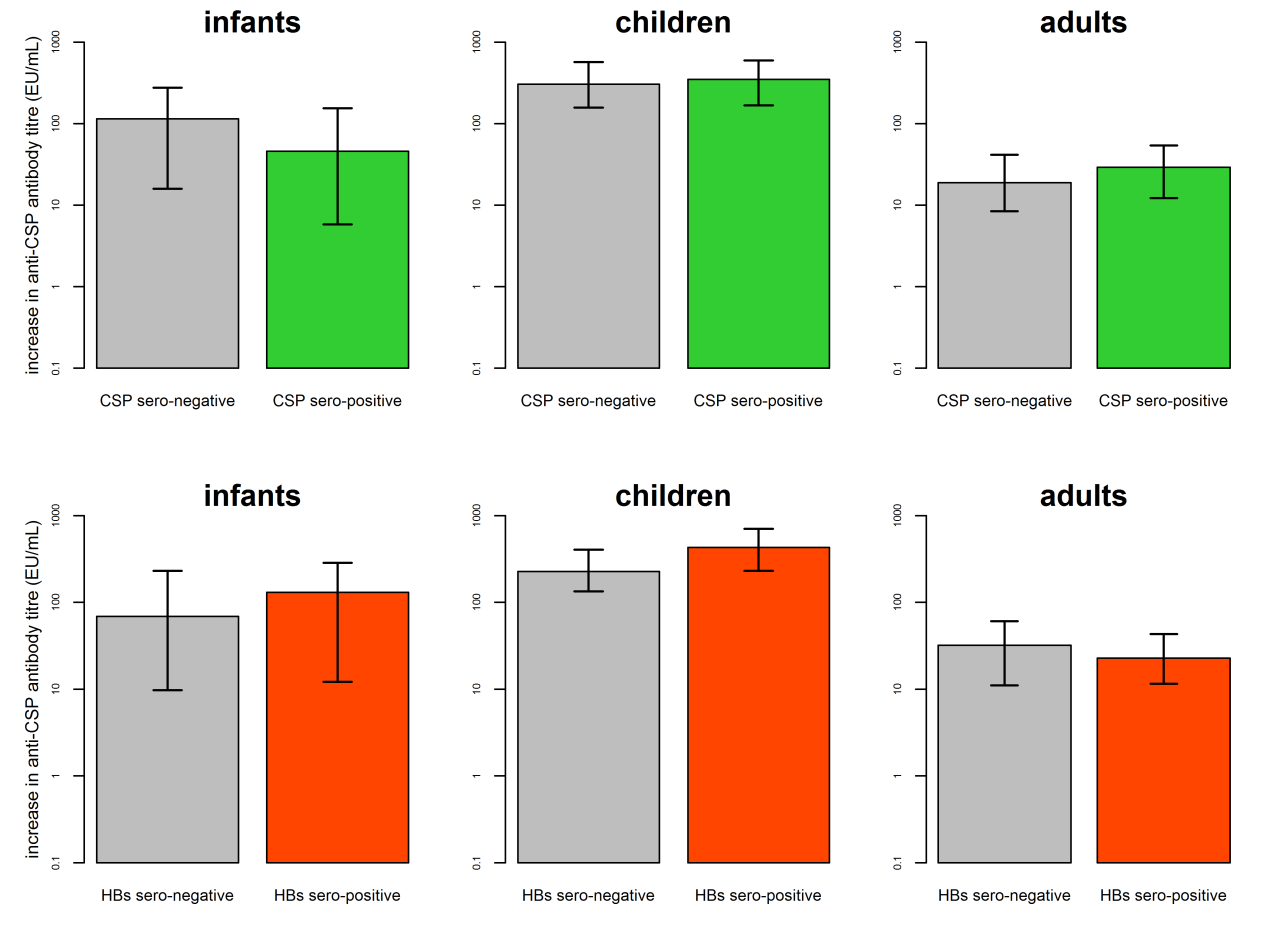
**

Figure S2: Effect of anti-CSP and anti-HBs antibody sero-positivity on median anti-CSP antibody titre following RTS,S vaccination. Coloured bars represent median increase in anti-CSP antibody titre following vaccination and the black bars represent the interquartile ranges. In all cases there is substantial overlap in the range of increase in anti-CSP antibody titres. There are statistically significant differences between the mean increase in anti-CSP antibody titres between: anti-CSP sero-negative and sero-positive infants; anti-CSP sero-negative and sero-positive adults; anti-HBs sero-negative and sero-positive children; and anti-HBs sero-negative and sero-positive adults.

Table 2 in the manuscript presents the results of linear regression models of the relationship between peak anti-CSP antibody titre following RTS,S vaccination ( log_10_(CSP_peak_) ) and underlying covariates. Only measurements of antibody titre in units of EU/mL were included in the regression analysis. Linear regression analyses were performed using the R statistical computing language [[1](,#_ENREF_1)]. Table S1 presents the same results as Table 2, but expressed in terms of the estimated percentage change in peak anti-CSP antibody titre following vaccination due to each of the examined covariates.

Both Model 1 and Model 2 incorporated trial site as a random effect to account for between site heterogeneity not captured by the fixed effects. The linear regression models presented in Table 2 are repeated in Table S2 below, with the exception that trial site is no longer included as a random effect. The most notable change is that co-administration of RTS,S with EPI vaccines is predicted to cause a statistically significant reduction in CSP_peak_ (P<0.001). The effect of site-level parasite prevalence on CSP_peak_ also becomes statistically significant (P<0.001), although this may be an artefact of the data attributable to the fact that all participants in a given trial site are assigned the same parasite prevalence.

Table S1: Estimates of the impact of covariates on peak anti-CSP antibody titre ( log_10_( CSP_peak_ / (EU/mL) ) ) following the final vaccine dose. Estimates are presented as percentage change in peak anti-CSP antibody titre compared to the baseline of RTS,S vaccination in a child receiving three doses of RTS,S/AS01 administered via a 0,1,2 month schedule without co-administration of other vaccines. ^¶^ indicates the percentage change associated with a log_10_ fold change in pre-vaccination antibody titre. † indicates the percentage change associated with a 1% change in parasite prevalence.

|  | **Model 1 (N=2659)** | |  | **Model 2 (N=1515)** | |
| --- | --- | --- | --- | --- | --- |
|  | Estimate (95% CI) | P value |  | Estimate (95% CI) | P value |
| RTS,S vaccination | 389 (186, 813) EU/mL | < 0.001 |  | 316 (132, 741) EU/mL | < 0.001 |
| Infants | -67%(-89%, -5%) | 0.04 |  | -61% (-88%, +23%) | 0.11 |
| Adults | -95% (-99%, -80%) | 0.002 |  | -93% (-99%, -67%) | 0.01 |
| log_10_(CSP_base_)*children ^¶^ | +12% (-9%, +38%) | 0.28 |  | -7% (-28, +23%) | 0.66 |
| log_10_(CSP_base_)*infants ^¶^ | -74% (-83%, -60%) | < 0.001 |  | -67% (-80%, -46%) | < 0.001 |
| log_10_(CSP_base_)*adults ^¶^ | +74% (+17%, +157%) | 0.006 |  | +100% (+29%, +202%) | 0.002 |
| adjuvant (AS02 *vs* AS01) | -26% (-37%, -11%) | < 0.001 |  | -24% (-35%, -11%) | < 0.001 |
| parasite prevalence† | +1% (-1%, +7%) | 0.32 |  | +1% (-1%, +8%) | 0.35 |
| doses (<3 *vs* 3) | -65% (-73%, -56%) | < 0.001 |  | -66% (-73%, -57%) | < 0.001 |
| schedule (017m *vs* 012m) | -86% (-88%, -83%) | < 0.001 |  | -86% (-88%, -83%) | < 0.001 |
| co-administration | -68% (-91%, +7%) | 0.095 |  | -70% (-92%, +17%) | 0.13 |
| log_10_(HBs_base_)*children ^¶^ | – | – |  | +12% (+5%, +23%) | 0.005 |
| log_10_(HBs_base_)*infants ^¶^ | – | – |  | -9% (-28%, +12%) | 0.34 |
| log_10_(HBs_base_)*adults ^¶^ | – | – |  | -24% (-37%, -9%) | 0.002 |

Table S2: Estimates of the impact of covariates on peak anti-CSP antibody titre ( log_10_( CSP_peak_ / (EU/mL) ) ) following the final vaccine dose. The RTS,S vaccine effect is the estimated peak anti-CSP antibody titre for a child receiving three doses of RTS,S/AS01 administered via a 0,1,2 month schedule without co-administration of other vaccines. Model 1 and Model 2 are as described in Table 2 in the manuscript except that trial site has not been included as a random effect.

|  | **Model 1 (N=2659)** | |  | **Model 2 (N=1515)** | |
| --- | --- | --- | --- | --- | --- |
|  | Estimate (95% CI) | P value |  | Estimate (95% CI) | P value |
| RTS,S vaccination | 2.61 (2.53, 2.68) | < 0.001 |  | 2.41 (2.29, 2.52) | < 0.001 |
| Infants | - 0.43(-0.58, -0.30) | < 0.001 |  | - 0.46 (-0.65, -0.27) | < 0.001 |
| Adults | - 1.22 (-1.33, -1.11) | < 0.001 |  | - 1.03 (-1.19, -0.88) | < 0.001 |
| log_10_(CSP_base_)*children | 0.05 (-0.05, 0.14) | 0.32 |  | - 0.02 (-0.14, 0.10) | 0.72 |
| log_10_(CSP_base_)*infants | - 0.74 (-0.93, -0.57) | < 0.001 |  | - 0.68 (-0.89, -0.47) | < 0.001 |
| log_10_(CSP_base_)*adults | 0.20 (0.02, 0.39) | 0.03 |  | 0.28 (0.08, 0.47) | 0.006 |
| adjuvant (AS02 *vs* AS01) | - 0.34 (-0.38, -0.29) | < 0.001 |  | - 0.21 (-0.28, -0.15) | < 0.001 |
| parasite prevalence | 0.30 (-0.29, 0.89) | < 0.001 |  | 0.29 (0.18, 0.40) | < 0.001 |
| doses (<3 *vs* 3) | 0.002 (-0.08, 0.08) | 0.95 |  | - 0.13 (-0.21, -0.04) | 0.005 |
| schedule (017m *vs* 012m) | - 0.59 (-0.66, -0.52) | < 0.001 |  | - 0.59 (-0.66, -0.52) | < 0.001 |
| co-administration | - 0.62 (-0.75, 0.50) | < 0.001 |  | - 0.59 (-0.74, -0.45) | < 0.001 |
| log_10_(HBs_base_)*children | – | – |  | 0.11 (0.07, 0.14) | < 0.001 |
| log_10_(HBs_base_)*infants | – | – |  | - 0.04 (-0.13, 0.06) | 0.42 |
| log_10_(HBs_base_)*adults | – | – |  | - 0.17 (-0.26, -0.09) | < 0.001 |

Table S3 shows the estimated peak anti-CSP antibody titre following vaccination with RTS,S/AS01 for cohorts of adults, children, infants, and infants co-administered with EPI vaccines as predicted by Model 1 in Table 2. The reference scenario for Model 1 predictions is for a case with log_10_(CSP_base_) = 0 and parasite prevalence = 5%. This is not representative of all of the cohorts of children and adults from phase 2 trials who had varying baseline anti-CSP antibody titres and were from sites with varying parasite prevalences. To account for this variation, estimates of CSP_peak_ adjusted for CSP_base_­ and parasite prevalence are also presented in Table S3.

Table S3: Estimated peak anti-CSP antibody titre following RTS,S vaccination. CSP_peak_ shows the mean and 95% range of the peak anti-CSP antibody titre following 3 doses of RTS,S/AS01 administered in a 0, 1, 2 month schedule in an individual without previous malaria exposure in a setting with 5% parasite prevalence. CSP­_peak_(adjusted) provides estimates adjusted for the range of baseline anti-CSP antibody titres and parasite prevalences observed in the phase 2 trial sites

| **Cohort** | **Adjuvant** | **CSP_peak_** | **CSP_peak_(adjusted)** |
| --- | --- | --- | --- |
| Adults | AS01 | 29 (3, 315) | 42 (4, 484) |
| Children | AS01 | 394 (36, 4291) | 465 (41, 5305) |
| Infants | AS01 | 311 (28, 3436) | 333 (29, 3847) |
| infants (co-ad) | AS01 | 74 (7, 812) | 82 (7, 941) |

In the linear regression models for the dependence of peak anti-CSP antibody titres on covariates, age was included as a categorical variable (infants <3 months; children >3 months and <5 years; and adults >18 years) to avoid making parametric assumptions about the relationship between age and CSP_peak_ given that the trials were restricted to these age categories. Table S4 presents the results of linear regression models focussing just on the children age category, with age in years included as a linear variable. For children between the ages of 3 months and 5 years, CSP_peak_ is predicted to decrease with increasing age (p < 0.0001). This suggests that younger children (e.g. < 2 years) had more immunogenic responses to RTS,S vaccination than older children (e.g. > 2 years).

Table S4: Estimates of covariates associated with peak anti-CSP antibody titre ( log_10_( CSP_peak_ / (EU/mL) ) ) following the final vaccine dose in children between the ages of 3 months and 5 years.

|  | **Model 1 (N=1939)** | |  | **Model 2 (N=1016)** | |
| --- | --- | --- | --- | --- | --- |
|  | Estimate (95% CI) | P value |  | Estimate (95% CI) | P value |
| RTS,S vaccination | 2.81 (2.69, 2.93) | < 0.001 |  | 2.65 (2.51, 2.79) | < 0.001 |
| age | -0.12 (-0.16, -0.09) | < 0.001 |  | -0.09 (-0.14, -0.04) | < 0.001 |
| log_10_(CSP_base_) | 0.13 (-0.03, 0.29) | 0.12 |  | 0.06 (-0.10, 0.22) | 0.45 |
| log_10_(CSP_base_)*age | -0.02 (-0.08, 0.04) | 0.45 |  | -0.02 (-0.09, 0.05) | 0.57 |
| adjuvant (AS02 *vs* AS01) | -0.18 (-0.24, -0.12) | < 0.001 |  | -0.14 (-0.19, -0.08) | 0.001 |
| parasite prevalence | 0.19 (0.02, 0.37) | 0.03 |  | 0.18 (0.09, 0.28) | < 0.001 |
| doses (<3 *vs* 3) | -0.09 (-0.18, -0.01) | 0.04 |  | -0.13 (-0.20, -0.06) | < 0.001 |
| schedule (017m *vs* 012m) | -0.20 (-0.29, -0.11) | < 0.001 |  | -0.24 (-0.31, -0.16) | < 0.001 |
| log_10_(HBs_base_) | – | – |  | 0.09 (0.04, 0.15) | < 0.001 |
| log_10_(HBs_base_)*age | – | – |  | -0.03 (-0.06, -0.01) | 0.003 |

The peak anti-CSP antibody titres in unvaccinated participants following the final dose of the control vaccine were analysed to test for associations with a number of covariates. Table S5 presents the results for control participants of the same linear regression models for vaccinated participants detailed in Table 2 of the main manuscript. Anti-CSP antibody titre following control vaccination was not associated with number of doses, schedule, co-administration status or baseline anti-HBs antibody titres. Anti-CSP antibody titre following control vaccination was significantly associated with age category, with adults having the highest antibody titres and infants the lowest, most likely due to the age-dependent natural acquisition of antibodies with exposure.

Table S5: Estimates of the impact of covariates on peak anti-CSP antibody titre ( log_10_( CSP_peak_ / (EU/mL) ) ) following the final dose of control vaccine. The control vaccine effect is the estimated peak anti-CSP antibody titre for a child receiving three doses of control vaccine administered via a 0,1,2 month schedule without co-administration of other vaccines. Model 1 and Model 2 are as described in Table 2 of the main manuscript.

|  | **Model 1 (N=1943)** | |  | **Model 2 (N=821)** | |
| --- | --- | --- | --- | --- | --- |
|  | Estimate (95% CI) | P value |  | Estimate (95% CI) | P value |
| Control vaccination | -0.68 (-0.83, -0.53) | < 0.001 |  | -0.48 (-0.77, -0.19) | 0.001 |
| Infants | -0.71(-0.92, -0.49) | < 0.001 |  | -0.78 (-1.16, -0.39) | 0.01 |
| Adults | 0.32 (0.14, 0.49) | 0.008 |  | 0.17 (-0.10, 0.44) | 0.29 |
| log_10_(CSP_base_)*children | 0.52 (0.46, 0.58) | < 0.001 |  | 0.54 (0.44, 0.63) | < 0.001 |
| log_10_(CSP_base_)*infants | -0.56 (-0.67, -0.44) | < 0.001 |  | -0.55 (-0.71, -0.39) | < 0.001 |
| log_10_(CSP_base_)*adults | 0.39 (0.29, 0.50) | < 0.001 |  | 0.38 (0.25, 0.50) | < 0.001 |
| parasite prevalence† | 0.42 (0.15, 0.69) | 0.002 |  | 0.29 (-0.08, 0.65) | 0.13 |
| doses (<3 *vs* 3) | -0.03 (-0.80, 0.74) | 0.89 |  | -0.09 (-0.85, -0.66) | 0.80 |
| schedule (017m *vs* 012m) | 0.03 (-0.29, 0.34) | 0.87 |  | -0.13 (-0.56, 0.31) | 0.59 |
| co-administration | -0.10 (-0.32, 0.13) | 0.42 |  | – | – |
| log_10_(HBs_base_)*children | – | – |  | -0.02 (-0.06, 0.01) | 0.22 |
| log_10_(HBs_base_)*infants | – | – |  | 0.009 (-0.07, 0.08) | 0.81 |
| log_10_(HBs_base_)*adults | – | – |  | 0.04 (-0.01, 0.09) | 0.16 |

1. **Statistical methods for antibody decay models**

Following vaccination with RTS,S, anti-CSP antibody titres are assumed to increase to CSP_peak_ and then decay over time *t* according to a bi-phasic exponential model as follows:

$$CSP(t)=CSP_{\text{peak}}\left(\rho e^{-\log(2)\frac{t}{d_{s}}}+(1-\rho)e^{-\log(2)\frac{t}{d_{l}}}\right)$$

where *d_s_* and *d_l_* are the half-lives of the short-lived and long-lived components of the antibody response, and *ρ* is the proportion of the antibody response that is short-lived. Models with a single phase of exponential decay were also tested, but these gave a poor fit to the data.

**Model fitting**

The model was fitted to longitudinal antibody titre measurements from vaccinated participants in each study site. Mixed effects methods were used to capture the natural variation in antibody dynamics between individual participants, whilst estimating the average value and variance of the immune parameters across the entire cohort of children [[2](#_ENREF_2), [3](#_ENREF_3)]. The models were fitted in a Bayesian framework using Markov Chain Monte Carlo methods. Mixed effects methods allow local parameters to be estimated for each child individually, with these local (or mixed effects) parameters being drawn from global distributions[[2](#_ENREF_2)]. For example, for each participant *n* the half-life of the short-lived component of the antibody response may be estimated as$d_{s}^{n}$ (an individual specific parameter). These *N* estimates of the local parameters $d_{s}^{n}$will be drawn from a probability distribution. A Log-Normal distribution is suitable as it has positive support on$[0,\infty)$. Thus we have$\log(d_{s}^{n})\sim N\left(\mu_{s},\sigma_{s}^{2}\right)$. The mean$d_{s}$and the variance$\Sigma_{s}^{2}$of the estimates of$d_{s}^{n}$are given by$d_{s}=e^{\mu_{s}+\frac{\sigma_{s}^{2}}{2}}$and$\Sigma_{s}^{2}=\left(e^{\sigma_{s}^{2}}-1\right)e^{2\mu_{s}+\sigma_{s}^{2}}$. The relationship between the global parameters describing the population level distribution and the local parameters for each individual in the population are depicted in the schematic diagram in Figure S3.


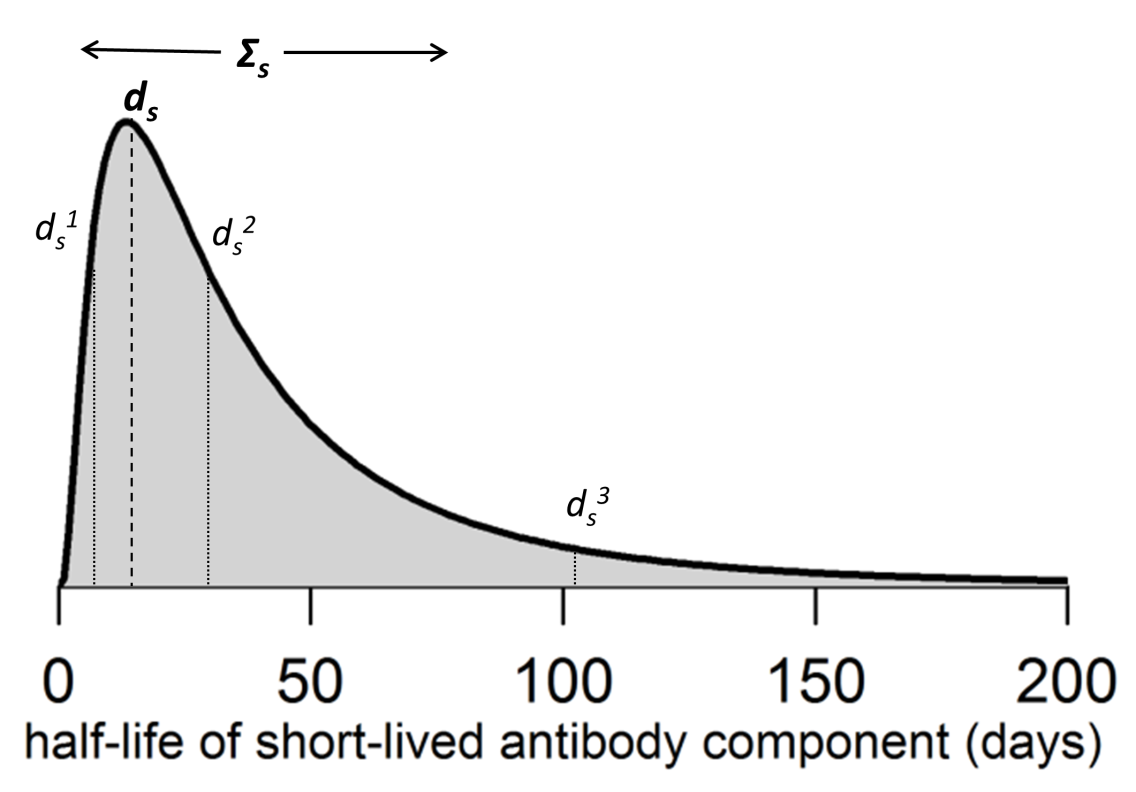


Figure S3: Schematic representation of the relationship between the global and local parameters for the half-life of the short-lived component of the RTS,S-induced antibody response. It is assumed that half-lives are log-Normally distributed throughout the population. The global parameters define the mean *d_s_* and standard deviation Σ_s_ of this distribution. The local parameters *d_s_^n^* for each of the *n* participants follow the log-Normal distribution defined by the global parameters. 3 representative local parameters are given for illustration.

**Model likelihood**

For participant *n* we have data on observed antibody titres $A^{n}=\left\{a_{1},...,a_{J}\right\}$at times$T^{n}=\left\{t_{1},...,t_{J}\right\}$. We denote $D^{n}=\left(A^{n},T^{n}\right)$to be the vector of data for participant *n*. For participant *n*, the three parameters $d_{s}^{n}$ , $d_{l}^{n}$ and $\rho^{n}$ are estimated. These parameters are denoted$\theta^{n}=\left(d_{s}^{n},d_{l}^{n},\rho^{n}\right)$. The model predicted antibody titres will be$\left\{CSP(t_{1}),CSP(t_{2}),...,CSP(t_{J})\right\}$. We assume Normally distributed measurement error such that the difference between the observed antibody titre$a_{j}$and the predicted antibody titre$CSP(t_{j})$will be Normally distributed with variance$\sigma_{\text{obs}}^{2}$, truncated at zero as negative antibody titres are not allowed. For model predicted antibody titres $CSP(t_{j})$the data likelihood for participant *n* is given by

$$L_{\text{mod}}^{n}\left(\theta^{n}\left|D^{n}\right.\right)=\prod_{j\in J} \frac{1}{\sigma_{\text{obs}}\sqrt{2\pi}}\frac{e^{-\frac{\left(CSP(t_{j})-a_{j}\right)^{2}}{2\sigma_{\text{obs}}^{2}}}}{1-\Phi\left(0\left|CSP(t_{j}),\sigma_{\text{obs}}^{2}\right.\right)}$$

$\Phi$is the cumulative normal distribution (i.e. a normalizing factor), with$1-\Phi\left(0\left|CSP(t_{j}),\sigma_{\text{obs}}^{2}\right.\right)$ being the area under a normal curve with mean$CSP(t_{j})$and variance $\sigma_{\text{obs}}^{2}$in the region $[0,\infty)$.

**Mixed effects likelihood**

There are three mixed effects parameters to be estimated: $d_{s}$, $d_{l}$ and $\rho$and the mixed effects likelihood is thus

$$L_{\text{mix}}^{n}\left(\theta^{n}\left|D^{n}\right.\right)=\frac{e^{-\frac{(\log(d_{s}^{n})-\mu_{s})^{2}}{2\sigma_{s}^{2}}}e^{-\frac{(\log(d_{l}^{n})-\mu_{l})^{2}}{2\sigma_{l}^{2}}}e^{-\frac{(\log(\frac{\rho^{n}}{1-\rho^{n}})-\mu_{\rho})^{2}}{2\sigma_{\rho}^{2}}}}{\left(2\pi\right)^{\frac{3}{2}}d_{s}^{n}d_{l}^{n}\sigma_{s}\sigma_{l}\rho^{n}(1-\rho^{n})\sigma_{\rho}}L_{\text{mod}}^{n}\left(\theta^{n}\left|D^{n}\right.\right)$$

As the proportion of the antibody response that is short-lived must be bounded by 0 and 1, the local parameters $\rho^{n}$are assumed to be drawn from a Logit-Normal distribution with mean$\mu_{\rho}$ and variance$\sigma_{\rho}^{2}$, i.e. $\log(\frac{\rho^{n}}{1-\rho^{n}})\sim N\left(\mu_{\rho},\sigma_{\rho}^{2}\right)$

**Total model likelihood**

Denote$D=\left\{D^{1},...,D^{N}\right\}$to be the vector of data for all *N* participants. We denote $\theta=\left(d_{s},d_{l},\rho,\sigma_{s},\sigma_{l},\sigma_{\rho},\sigma_{\text{obs}},\theta^{1},...,\theta^{N}\right)$ to be the combined vector of global parameters and local parameters to be estimated. The total likelihood is obtained by multiplying the likelihood for each child

$$L_{\text{total}}\left(\theta\left|D\right.\right)=\prod_{n\in N} L_{\text{mix}}^{n}\left(\theta^{n}\left|D^{n}\right.\right)$$

**Markov Chain Monte Carlo parameter update**

The model was fitted to the data using Markov Chain Monte Carlo (MCMC) methods. Parameters were updated at each MCMC iteration using a random walk Metropolis-Hastings algorithm with two update stages illustrated below. A ′ indicates an attempted update parameter.

1. **Local parameter update.**  For each participant *n*:

- Update local parameters in one step:
- Calculate updated mixed effects likelihood $L_{\text{mix}}^{n}\left(\theta_{n}^{'}\left|D_{n}\right.\right)$
- Accept the parameter update with probability $\min\left(1,\frac{L_{\text{mix}}^{n}\left(\theta_{n}^{'}\left|D_{n}\right.\right)}{L_{\text{mix}}^{n}\left(\theta_{n}\left|D_{n}\right.\right)}\right)$

1. **Global parameter update.**

- Update global parameters in one step: $\theta'=\left(d_{s}^{'},d_{l}^{'},\rho^{'},\sigma_{s}^{'},\sigma_{l}^{'},\sigma_{\rho}^{'},\sigma_{\text{obs}}^{'},\theta^{1},...,\theta^{N}\right)$
- Calculate updated total likelihood $L_{\text{total}}\left(\theta'\left|D\right.\right)$and the updated prior likelihood$P\left(\theta'\right)$
- Accept the parameter update with probability $\min\left(1,\frac{L_{\text{total}}\left(\theta'\left|D\right.\right)P\left(\theta'\right)}{L_{\text{total}}\left(\theta\left|D\right.\right)P\left(\theta\right)}\right)$

All updates were attempted with Normal proposal distributions. The MCMC algorithm was implemented in C++. The variances of the proposal distributions were first estimated by performing 1 million MCMC iterations to estimate the variance of the posterior distributions. Uninformative uniform priors were placed on global parameters. 20 million MCMC iterations were computed with calibration of acceptance rates using a Robbins-Munro algorithm[[4](#_ENREF_4)]. All Markov chains were visually examined for appropriate mixing and convergence. Such large numbers of iterations were needed because of the large number of parameters to be estimated. The effective number of iterations was calculated using the effectiveSize routine in the R library coda [[5](#_ENREF_5)] and the effective size was checked to be > 10,000 in all cases. The MCMC fitting process was repeated multiple times to ensure consistent results and test for lack of convergence.

Table 65 shows the estimated parameters for two variants of the model. Model *a* assumes that the dynamics of both RTS,S/AS01 and RTS,S/AS02 induced anti-CSP antibody titres are identical. Model *b* assumes that the dynamics of RTS,S/AS02 and RTS,S/AS01 induced antibodies are described by different parameters with the exception that the half-life of the long-lived component of vaccine-induced antibodies is equal for the two adjuvants. For reasons of parsimony, Model *a* is chosen.

Table S6: Estimates of parameters describing the dynamics of RTS,S induced anti-CSP antibodies for mixed effects models. Model *a* assumes that the dynamics of both RTS,S/AS01 and RTS,S/AS02 induced anti-CSP antibody titres are identical. Model *b* assumes that the dynamics of RTS,S/AS02 and RTS,S/AS01 induced antibodies are described by different parameters with the exception that the half-life of the long-lived component of vaccine-induced antibodies is equal for the two adjuvants. U denotes a uniform distribution.

|  |  |  | **Model *a*** | **Model *b*** |
| --- | --- | --- | --- | --- |
|  | **parameter** | **prior distribution** | **posterior median and  credible intervals** | **posterior median and  credible intervals** |
| *d_s_*_,AS01_ | half-life of short-lived component (AS01) | U(0, 5000) | 46 (43 – 49) days | 43 (39 – 47) days |
| *d_s_*_,AS02_ | half-life of short-lived component (AS02) | U(0, 5000) | 46 (43 – 49) days | 47 (43 – 52) days |
| *d_l_*_,AS01_ | half-life of long-lived component (AS01) | U(0, 50000) | 594 (551 – 645) days | 582 (540 – 630) days |
| *d_l_*_,AS02_ | half-life of long-lived component (AS02) | U(0, 50000) | 594 (551 – 645) days | 582 (540 – 630) days |
| ρ_AS01_ | proportion of short-lived antibodies (AS01) | U(0, 1) | 82% (80% - 83%) | 84% (83% – 85%) |
| ρ_AS02_ | proportion of short-lived antibodies (AS02) | U(0, 1) | 82% (80% - 83%) | 80% (78% – 81%) |
| *σ_s_*_,AS01_ | standard deviation in half-life of short-lived component (AS01) | U(0, 5000) | 52 (48 – 60) days | 50 (41 – 62) days |
| *σ_s_*_,AS02_ | standard deviation in half-life of short-lived component (AS02) | U(0, 5000) | 52 (48 – 60) days | 53 (45 – 64) days |
| *σ_l_*_,AS01_ | standard deviation in half-life of long-lived component (AS01) | U(0, 5000) | 377 (323 – 447) days | 362 (311 – 427) days |
| *σ_l_*_,AS02_ | standard deviation in half-life of long-lived component (AS02) | U(0, 5000) | 377 (323 – 447) days | 362 (311 – 427) days |
| *σ*_ρ,AS01_ | standard deviation in proportion short-lived antibodies (AS01) | U(0, 5000) | 0.12 (0.11 – 0.13) | 0.10 (0.09 – 0.12) |
| *σ*_ρ,AS02_ | standard deviation in proportion short-lived antibodies (AS02) | U(0, 5000) | 0.12 (0.11 – 0.13) | 0.13 (0.12 – 0.4) |
| σ_obs_ | observational variance | U(0, 5000) | 9.7 (9.5 – 9.9) EU/mL | 9.7 (9.5 – 9.9) EU/mL |

1. **Statistical methods for survival analysis**

For each vaccinated participant, anti-CSP antibody titres CSP(*t*) were calculated using equation (1) and the estimated individual-level parameters$\left(d_{s}^{n},d_{l}^{n},\rho^{n}\right)$ . We used the model-predicted anti-CSP antibody titres over time to estimate the relationship between antibody titres and protection. If a vaccinated participant is challenged with infection at time *t* by an infectious mosquito, the probability that the infection will be prevented by RTS,S induced antibodies, denoted *V(t)*, can be described by the following dose-response curve:

where *V*_max_, α and β are parameters to be estimated. The scale parameter β is assumed to be equal for infants (≤3 months) and children (>3months and <5 years), but different for adults. The shape parameter α is equal across all age groups. In all but one trial, anti-CSP antibody titres were measured in ELISA units per millilitre (EU/mL). In one trial of RTS,S/AS02 in Gambia adults[[6](#_ENREF_6)], anti-CSP antibody titres were measured in units of micrograms per millilitre (µg/mL). Separate shape and scale parameters were fitted to the data from this trial. The estimated functional forms for *V*(*t*) are plotted in Figure 2a-c of the main manuscript.

We assume that each participant *n* is subjected to a fixed entomological inoculation rate depending on their trial site, denoted *EIR^n^*. A participant’s exposure to infectious bites will depend on their age *a* in years. Young children are assumed to receive less mosquito bites than adults due their smaller body sizes and tendency to spend more time indoors at night time[[7](#_ENREF_7), [8](#_ENREF_8)]. The rate at which a trial participant of age *a* is exposed to infectious bites is thus dependent on their age and trial site and can then be modelled as:

$$\varepsilon^{n}(a)=EIR^{n}\underbrace{\left(1-\rho_{a}e^{-a/a_{0}}\right)}_{\text{age dependency}}$$

The model does not capture the effects of seasonal variations in transmission intensity, a potential limitation given the time scale of decay of the vaccine. The probability that a bite from an infectious mosquito progresses to blood-stage infection in an unvaccinated participant can be described by the previously described functional form [[9](#_ENREF_9)]:

$$b(t)=b_{0}\left(b_{1}+\frac{1-b_{1}}{1+\left(I_{B}(t)/I_{B0}\right)^{\kappa_{B}}}\right)$$

where *I_B_*(*t*) is the time-dependent immunity against infection, and *b*_0_, *b*_1_, *I_B_*_0_ and *κ_B_*  are immune parameters defined in Table S7. The probability of infection per mosquito bite *b*(*t*) is assumed to decrease with increasing levels of infection-blocking immunity$I_{B}(\mathrm{t})$ , however this is assumed to be independent of RTS,S induced anti-CSP antibody titres CSP(*t*). The time-dependent hazard of infection on an unvaccinated trial participant is then given by:

$$\Lambda^{n}(t)=\varepsilon^{n}(a+t)b(t)$$

And the time-dependent hazard of infection on a vaccinated trial participant by

$$\Lambda^{n}(t)=\varepsilon^{n}(a+t)b(t)\left(1-V(t)\right)$$

Using a similar functional form we can calculate the probability that an infection leads to an episode of clinical malaria[[9](#_ENREF_9)]

where *I_CA_*(*t*) is the time-dependent immunity against clinical malaria, *I_CM_*(*t*) the time-dependent maternal immunity against clinical malaria, and φ_0_, φ_1_, *I_C_*_0_ and *κ_C_* are immune parameters defined in Table S7. The time dependent hazard of an episode of clinical malaria in an unvaccinated trial participant can be modelled as

And the time-dependent hazard of an episode of clinical malaria in a vaccinated trial participant is given by

The rate at which episodes of clinical malaria were detected in trials was reduced by a further factor *r*_ACD_ or *r*_PCD_ due to imperfect detection according to whether the trial implemented active case detection (ACD) or passive case detection (PCD). *r*_ACD_ and *r*_PCD_ were fixed at the values estimated in Griffin *et al* [[9](#_ENREF_9)] (see Table S7).

The acquisition of immunity against infection ( *I_B_* ) and clinical malaria ( *I_CA_* ) is described by the following set of differential equations

$$\begin{matrix} \frac{\partial I_{B}}{\partial t}+\frac{\partial I_{B}}{\partial a}=\frac{\varepsilon(t)}{\varepsilon(t)u_{B}+1}-\frac{I_{B}}{d_{B}} \\ \frac{\partial I_{CA}}{\partial t}+\frac{\partial I_{CA}}{\partial a}=\frac{\Lambda(t)}{\Lambda(t)u_{C}+1}-\frac{I_{CA}}{d_{C}} \end{matrix}$$

where *u_B_*, *d_B_*, *u_C_* and *d_C_* are immune parameters as defined in Table S6.Maternal clinical immunity *I_CM_* is assumed to be at birth a proportion *P_M_* of the acquired immunity of a 20 year old and to decay at rate 1/*d_M_*.

$$I_{CM}(a)=P_{M}I_{CA}(20)e^{-\frac{a}{d_{M}}}$$

**Model likelihood**

Using the hazards described above a survival model was fitted to the time of first infection (for ACDi trials) or times of all clinical episodes (for ACDc and PCD trials). One trial in Ilha Josina cohort 2, Mozambique[[10](#_ENREF_10), [11](#_ENREF_11)] monitored for parasitological infection in the first 6 months after vaccination, and then switched to passive case detection for clinical malaria for the next 12 months. We assume a constant EIR throughout both periods of this trial.

During a vaccine trial, a participant *n* will avoid infection (*I^n^* =0) or become infected (*I^n^* = 1). Denote *τ*^n^ to be the time of infection, or if uninfected the end of follow up. The probability that participant *n* subjected to time-dependent exposure to malaria Λ^n^(*t*) will avoid infection (*I^n^* =0) until time *τ*^n^ is:

$$P\left(I^{n}=0\right)=e^{-\int_{0}^{\tau^{n}} \Lambda^{n}(t)dt}$$

The probability that participant *n* becomes infected (*I^n^* =1) at time *τ*^n^ is:

$$P\left(I^{n}=1\right)=\Lambda^{n}(t)e^{-\int_{0}^{\tau^{n}} \Lambda^{n}(t)dt}$$

Equations (15) and (16) can be combined to produce the likelihood that a model of time-dependent Λ^n^(*t*) fits the data as follows:

$$L_{\text{inf}}^{n}\left(\left.EIR^{n},\mathrm{k},\alpha,\beta,V_{\max}\right|I^{n},\tau^{n}\right)=\Lambda^{n}(\tau^{n})^{I^{n}}e^{-\int_{0}^{\tau^{n}} \Lambda^{n}(t)dt}$$

We can incorporate heterogeneity in exposure to infectious bites using a Gamma distribution with shape parameter *k* so that the mean number of infectious bites per day is *EIR^n^* with variance (*EIR^n^*)2/*k*. If we separate the force of infection into constant *EIR^n^* and the time varying component *f*(*t*), such that *Λ^n^*(*t*)=*EIR^n^* *f*(*t*) then the likelihood can be written as:

$$L_{\text{inf}}^{n}\left(\left.EIR^{n},\mathrm{k},\alpha,\beta,V_{\max}\right|I^{n},\tau^{n}\right)=\int_{0}^{\infty} \left(\left(xf(\tau^{n})\right)^{I^{n}}e^{-\int_{0}^{\tau^{n}} xf(t)dt}\right)\Gamma_{d}\left(x\left|EIR^{n},k\right.\right)dx$$

where Γ*_d_*(*x*|*EIR^n^*,*k*) is a Gamma distribution with mean *EIR^n^* and shape parameter *k* defined as follows:

$$\Gamma_{d}\left(x\left|EIR^{n},k\right.\right)=\left(\frac{k}{EIR^{n}}\right)^{k}\frac{x^{k-1}e^{-\frac{kx}{EIR^{n}}}}{\Gamma(k)}$$

Note that the subscript *d* denotes the Gamma distribution as opposed to the Gamma function. Equation (18) can be evaluated numerically using Gaussian quadrature with weights (*w_j_*) and abscissas (*x_j_*) derived from Gauss-Laguerre polynomials such that

$$L_{\text{inf}}^{n}\left(\left.EIR^{n},\mathrm{k},\alpha,\beta,V_{\max}\right|I^{n},\tau^{n}\right)=\sum_{j=1}^{J} w_{j}\left(\left(x_{j}f(\tau^{n})\right)^{I^{n}}e^{-\int_{0}^{\tau^{n}} x_{j}f(t)dt}\right)$$

The total likelihood for all *N*_inf_ participants with parasitological infection as an endpoint can be obtained by multiplying each participant’s likelihood in equation (18):

$$L_{\text{inf}}=\prod_{n=1}^{N_{\text{inf}}} L_{\text{inf}}^{n}$$

A participant *n* in a vaccine trial with clinical malaria as an endpoint will be followed up for time *T^n^* and experience *I^n^* episodes of clinical malaria at times$\{\tau_{1}^{n},...,\tau_{I^{n}}^{n}\}$. The likelihood that a model of time-dependent *H^n^*(*t*) fits the data is given by

$$L_{\text{clin}}^{n}\left(\left.EIR^{n},\mathrm{k},\alpha,\beta,V_{\max}\right|I^{n},\tau^{n}\right)=\left(\prod_{i=1}^{I^{n}} H^{n}\left(\tau_{i}^{n}\right)\right)e^{-\int_{0}^{T^{n}} H^{n}(t)dt}$$

Heterogeneity in exposure can be incorporated in the same manner as above to give

$$L_{\text{clin}}^{n}\left(\left.EIR^{n},\mathrm{k},\alpha,\beta,V_{\max}\right|I^{n},\tau^{n}\right)=\int_{0}^{\infty} \left(\left(\prod_{i=1}^{I^{n}} H^{n}\left(\tau_{i}^{n}\right)\right)e^{-\int_{0}^{T^{n}} H^{n}(t)dt}\right)\Gamma_{d}\left(x\left|EIR^{n},k\right.\right)dx$$

which can be evaluated numerically using Gaussian quadrature. The total likelihood for all *N*_clin_ participants with episodes of clinical malaria as an endpoint can be obtained by multiplying each participant’s likelihood in equation (23):

$$L_{\text{clin}}=\prod_{n=1}^{N_{\text{clin}}} L_{\text{clin}}^{n}$$

Finally, the model was fitted to all data from trial participants followed for parasitological infection and clinical malaria together, to give a total likelihood of:

$$L=L_{\text{inf}}L_{\text{clin}}$$

In the model likelihood described above episodes of clinical malaria are assumed to be due to variation in expsoure, an individual’s level of naturally-acquired immunity, and an individual’s vaccination status. After accounting for these factors, episodes of clinical malaria within an individual are assumed to be independent, e.g. an individual’s likelihood of having an episode of clinical malaria does not depend on whether they previosuly had malaria. Notably, there was substantial heterogeneity in the incidence of episodes of clinical malaria. Figure S4 shows the distribution of numbers of episodes of clinical malaria throughout the unvaccinated control cohort.


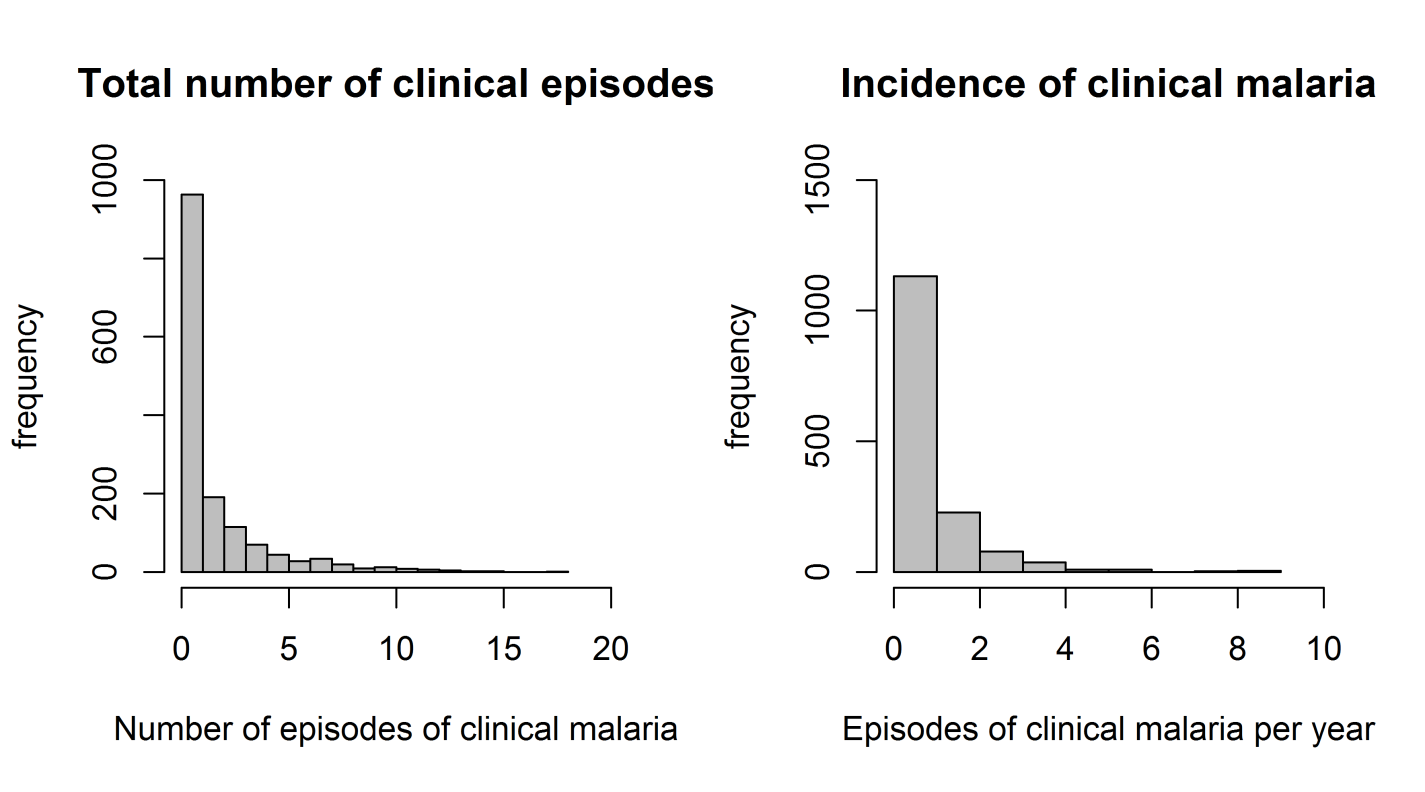


Figure S4: Distributions of total number of episodes of clinical malaria per person and incidence of clinical malaria (recorded as number of detected cases per year). Data are for infants and children in the unvaccinated control arm of trials where monitoring for episodes of clinical malaria was implemented using active or passive case detection.

The model was fitted to the combined data on infection and clinical malaria in a Bayesian framework using Markov Chain Monte Carlo methods. The posterior medians and 95% credible intervals for each of the estimated parameters are presented in Table S8.

The MCMC algorithm was implemented in C++. The variances of the proposal distributions were first estimated by performing 10,000 MCMC iterations to estimate the variance of the posterior distributions. Details of prior distributions are given in Table S8. 100,000 MCMC iterations were computed with calibration of acceptance rates using a Robbins-Munro algorithm[[4](#_ENREF_4)]. All Markov chains were visually examined for appropriate mixing and convergence. The MCMC fitting process was repeated multiple times to ensure consistent results and test for lack of convergence.

Table S8 contains estimates of the parameters describing the dose-response curve in equation (5): β, α and *V*_max_. Equation (5) can be re-arranged to obtain the anti-CSP antibody titre required for a given vaccine efficacy. For example, we can estimate *CSP_1/2_,* the antibody titre required for 50% efficacy against infection as follows:

$$CSP_{\frac{1}{2}}=\beta\left(\frac{0.5}{V_{\max}-0.5}\right)^{\frac{1}{\alpha}}$$

Evaluating equation (26) we find that an anti-CSP antibody titre of 51 (95% CrI:29 – 85) EU/mL is estimated to provide 50% vaccine efficacy against infection in children and infants. For adults, we estimate that an anti-CSP antibody titre of 19 (95% CrI: 4 – 83) EU/mL is required to provide 50% vaccine efficacy.

Table S7: Parameters describing the acquisition of natural immunity against *P. falciparum* infection and episodes of clinical malaria. All parameters are taken from ‘Estimates of the changing age-burden of *P. falciparum* malaria disease in sub-Saharan Africa’ by Griffin, Ferguson and Ghani[[9](#_ENREF_9)]. Estimated parameters are presented with 95% credible intervals.

| **Parameter description** | **Symbol** | **Value** | **95% credible interval** |
| --- | --- | --- | --- |
| **Age-dependent exposure** |  |  |  |
| Age-dependent biting parameter | *ρ_a_* | 0.85 | Fixed |
| Age-dependent parameter | *a*_0_ | 8 years | Fixed |
|  |  |  |  |
| **Immunity reducing probability of infection** |  |  |  |
| Probability with no immunity | *b_0_* | 0.590 | (0.389, 0.845) |
| Maximum relative reduction | *b_1_* | 0.5 | Fixed |
| Inverse of decay rate | *d_B_* | 10 years | Fixed |
| Scale parameter | *I_B0_* | 43.879 | (20.1, 120) |
| Shape parameter | *κ_B_* | 2.155 | (1.22, 2.93) |
| Duration in which immunity is not boosted | *u_B_* | 7.199 days | (2.63, 15.0) days |
|  |  |  |  |
| **Immunity reducing probability of clinical disease** |  |  |  |
| Probability with no immunity | *Φ_0_* | 0.792 | (0.548, 0.961) |
| Maximum relative reduction | *Φ_1_* | 0.0007 | (0.00005, 0.0025) |
| Inverse of decay rate | *d_C_* | 30 years | Fixed |
| Scale parameter | *I_C0_* | 18.024 | (11.9, 26.7) |
| Shape parameter | *κ_C_* | 2.369 | (1.99, 2.86) |
| Duration in which immunity is not boosted | *u_C_* | 6.063 days | (2.82, 11.1) days |
| New-born immunity relative to mother’s | *P_M_* | 0.774 | (0.536, 0.981) |
| Inverse of decay rate of maternal immunity | *d_M_* | 67.695 days | (59.0, 79.4) days |
|  |  |  |  |
| **Case detection: recorded incidence relative to daily ACD** |  |  |  |
| Weekly ACD | *r_ACD_* | 0.724 | (0.460, 0.923) |
| PCD | *r_PCD_* | 0.342 | (0.120, 0.860) |

Table S8: Parameters estimated from Bayesian MCMC model. * Uninformative uniform priors in the range (0 – 5000) were assumed. Other parameters were given log-Normal prior distributions, except for *V*_max_ which was constrained on the interval [0,1] and hence given a beta prior distribution.

| **Parameter** | **Prior median and 95% credible interval** | **Posterior median and 95% credible interval** | **Reported value** | **Source** |
| --- | --- | --- | --- | --- |
| EIR – Gambia | 25 (16 – 36) ibppy | 20 (16 – 24) ibppy | 1 – 50 ibppy | [[6](#_ENREF_6), [12](#_ENREF_12)] |
| EIR – Kisumu, Kenya | 20 (13 – 29) ibppy | 14 (11 – 19) ibppy | 31 ibppy | [[13](#_ENREF_13)] |
| EIR – Ilha Josina C2, Mozambique | 38 (25 – 55) ibppy | 20 (16 – 26) ibppy | 38 ibppy | [[10](#_ENREF_10), [14](#_ENREF_14)] |
| EIR – Mozambique infants | 38 (25 – 55) ibppy | 6 (5 – 8) ibppy | 38 ibppy | [[10](#_ENREF_10), [14](#_ENREF_14)] |
| EIR – Bagamoyo, Tanzania | 100 (66 – 145) ibppy | 60 (49 – 81) ibppy | 80 – 600 ibppy | [[13](#_ENREF_13), [15](#_ENREF_15)] |
| EIR – Manhica C1, Mozambique | 38 (25 – 55) ibppy | 22 (20 – 24) ibppy | 38 ibppy | [[10](#_ENREF_10)] |
| EIR – Kilifi, Kenya | 38 (34 – 42) ibppy | 32 (30 – 34) ibppy | 22 – 53 ibppy | [[16](#_ENREF_16)] |
| EIR – Korogwe, Tanzania | 90 (59 – 130) ibppy | 8 (6 – 10) ibppy | 90 ibppy | [[16](#_ENREF_16)] |
| EIR – Kintampo, Ghana | 250 (165 – 361) ibppy | 196 (135 – 275) ibppy | 269 ibppy | [[17](#_ENREF_17)] |
| EIR – Bagamoyo, Tanzania | 100 (66 – 145) ibppy | 44 (33 – 59) ibppy | 80 – 600 ibppy | [[13](#_ENREF_13)] |
| EIR – Lambaréné, Gabon | 23 (15 – 33) ibppy | 9 (7 – 12) ibppy | 23 ibppy | [[18](#_ENREF_18)] |
| gamma heterogeneity: *k* | 0.24 (0.16 – 0.35) | 0.49 (0.45 – 0.53) | – | [[19](#_ENREF_19)] |
| maximum efficacy: *V*_max_ | 90% (80% – 97%) | 88% (83% – 92%) | – | [[20](#_ENREF_20)] |
| dose-response: β (children & infants) | * | 32 (16 – 58) EU/mL | – |  |
| dose-response: β (adults) | * | 12 (3 – 48) EU/mL | – |  |
| dose-response: *α* | 1.74 (1.09 – 2.65) | 0.56 (0.42 – 0.73) | – | [[20](#_ENREF_20)] |
| dose-response: β (Gambian adults) | * | 11 (5 – 25) µg/mL | – |  |
| dose-response: *α* (Gambian adults) | 1.74 (1.09 – 2.65) | 1.63 (1.12 – 2.29) | – | [[20](#_ENREF_20)] |

The model for estimating the force of infection and dose-response and parameters in Table S8 was compared against two other models. Table S8 presents the results for Model 2 which has two scale parameters β for the dose-response curves, one for children & infants, and one for adults. We also consider Model 1 where the scale parameter β is the same for infants, children and adults. Similarly, we consider Model 3 which has three scale parameters β, one for each of infants, children and adults. Table S9 presents a comparison of these models using the Deviance Information Criterion (DIC) [21]. Of the three models, Model 2 had the smallest DIC and so was selected.

Table S9: Comparison of models for survival analysis using Deviance Information Criterion (DIC).

|  | **Model 1** | **Model 2** | **Model 3** |
| --- | --- | --- | --- |
| $\bar{L}$ | -39340.9 | -39337.7 | -39576.2 |
| $L\left(\bar{\theta}\right)$ | -39333.1 | -39330.2 | -39330.0 |
| $p_{D}$ | 15.7 | 14.9 | 15.6 |
| DIC | 78697.6 | 78690.2 | 78691.2 |
| ΔDIC | 7.4 | 0 | 1.0 |

1. **Uncertainty in estimated vaccine efficacy and cases averted**

Figure 3 in the main manuscript shows model estimated vaccine efficacy against infection and clinical malaria, and estimates of the cumulative numbers of cases of clinical malaria averted. Figure S5 shows the uncertainty associated with these estimates as represented by the 95% credible intervals for each estimated quantity. In addition to the uncertainty in the rate of decay of antibody titre and the association between antibody titre and vaccine efficacy captured in this analysis, there will be uncertainty in the model used for the rate of acquisition of natural immunity to clinical malaria[[9](#_ENREF_9)]. This model uncertainty in the rate of acquisition of immunity is not accounted for here, but is likely to substantially increase the degree of uncertainty in the model predictions.

In estimates of the cumulative number of cases of clinical malaria averted, we have assumed cases were detected via weekly active case detection. A trial with passive case detection would report substantially lower estimated numbers of cases averted (approximately 50% lower[[9](#_ENREF_9)]).

**
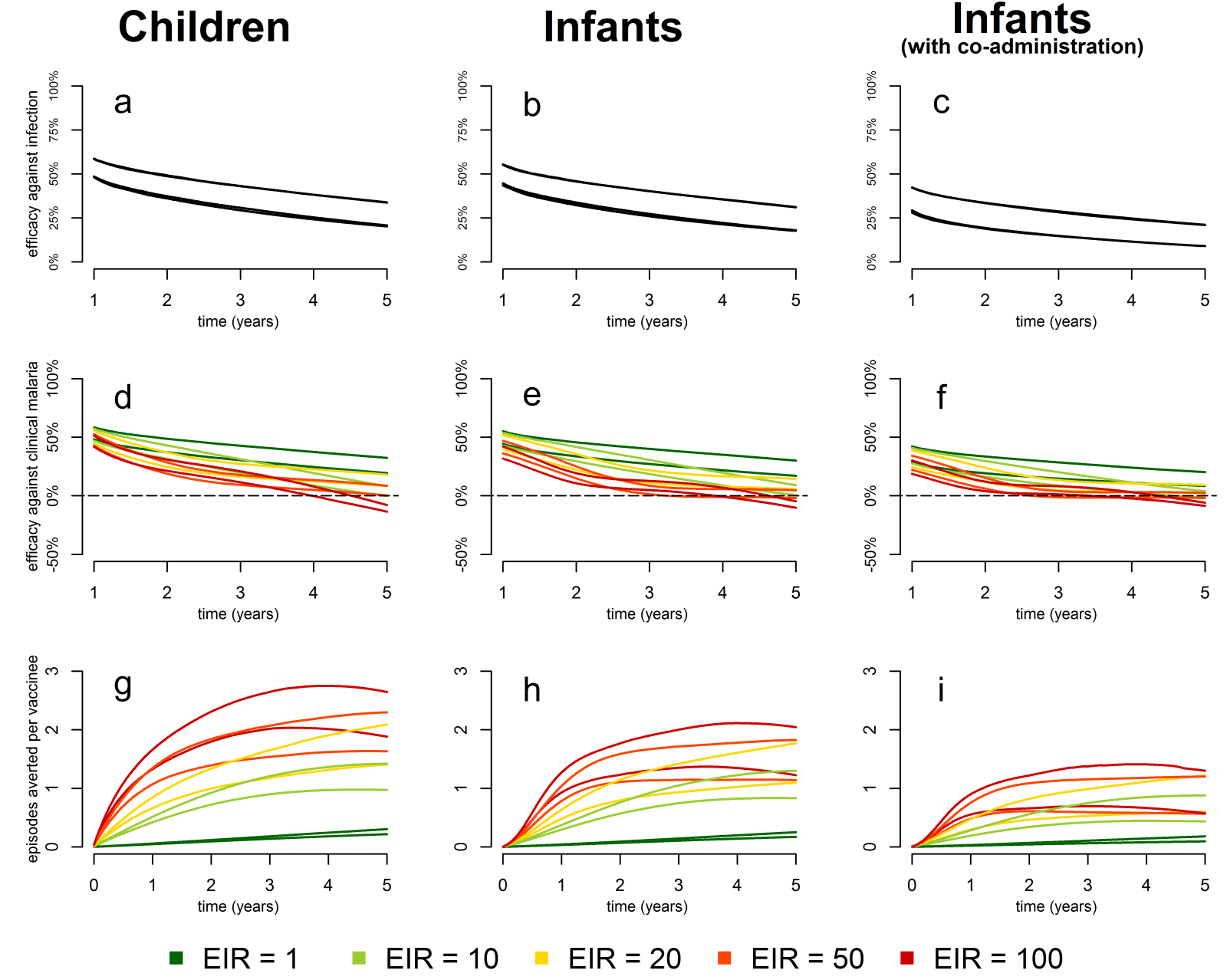
**

Figure S5: Uncertainty in estimates of vaccine efficacy and cumulative cases averted. Pairs of coloured lines represent the lower and upper 95% credible intervals for the estimated quantity. (a,b,c) Change in vaccine efficacy against infection over time for children (>3 months & <5 years), infants (≤3 months), and infants co-administered with EPI vaccines. Vaccine efficacy against infection at year x is the vaccine efficacy against all infections during the period (x-1,x) years. (d,e,f) Change in vaccine efficacy against episodes of clinical malaria over time for children, infants, and infants co-administered with EPI vaccines. Vaccine efficacy against clinical malaria at year x is the efficacy against all episodes during the period (x-1,x) years. (g,h,i) Cumulative episodes of clinical malaria averted by RTS,S per fully vaccinated child or infant. Cases averted at year x is the expected difference in the number of clinical episodes in the period (0,x) years between a vaccinated and unvaccinated child or infant.

**References**

1. R Core Team: **R: A language and environment for statistical computing.** Edited by Computing RFfS. Vienna, Austria; 2013.

2. Davidian M, Giltinan DM: **Nonlinear models for repeated measurement data: An overview and update**. *J Ag Bio Env Stat* 2003, **8**(4):387-419.

3. White M, Griffin JT, Akpogheneta OJ, Conway DJ, Koram KA, Riley E, Ghani AC: **Dynamics of the antibody response to *Plasmodium falciparum* infection in African children**. *J Inf Dis* 2014.

4. Atchade YF, Rosenthal JS: **On adaptive Markov chain Monte Carlo algorithms**. *Bernoulli* 2005, **11**(5):815-828.

5. Plummer M, Best NG, Cowles K, Vines K: **CODA: Convergence diagnosis and output analysis for MCMC**. In*.* <http://CRAN.R-project.org/Rnews;> 2006.

6. Bojang KA, Milligan PJM, Pinder M, Vigneron L, Alloueche A, Kester KE, Ballou WR, Conway DJ, Reece WHH, Gothard P *et al*: **Efficacy of RTS,S/ASO2 malaria vaccine against Plasmodium falciparum infection in semi-immune adult men in The Gambia: a randomised trial**. *Lancet* 2001, **358**(9297):1927-1934.

7. Carnevale P, Frezil JL, Bosseno MF, Pont FL, Lancien J: **Study of the aggressiveness of Anopheles gambiae A in relation to the age and sex of human subjects**. *Etude de l'agressivite d'Anopheles gambiae A en fonction et de l'age et du sexe des sujets humains* 1976:17 pp.

8. Port GR, Boreham PFL, Bryan JH: **The relationship of host size to feeding by mosquitos of the Anopheles-gambiae giles complex (Diptera, Culicidae)**. *Bull Entomol Res* 1980, **70**(1):133-144.

9. Griffin JT, Ferguson NM, Ghani AC: **Estimates of the changing age-burden of P. falciparum malaria disease in sub-Saharan Africa**. *Nat Comm* 2014, **5**.

10. Alonso PL, Sacarlal J, Aponte JJ, Leach A, Macete E, Milman J, Mandomando I, Spiessens B, Guinovart C, Espasa M *et al*: **Efficacy of the RTS,S/AS02A vaccine against Plasmodium falciparum infection and disease in young African children: randomised controlled trial**. *Lancet* 2004, **364**(9443):1411-1420.

11. Sacarlal J, Aide P, Aponte JJ, Renom M, Leach A, Mandomando I, Lievens M, Bassat Q, Lafuente S, Macete E *et al*: **Long-Term Safety and Efficacy of the RTS,S/AS02A Malaria Vaccine in Mozambican Children**. *J Inf Dis* 2009, **200**(3):329-336.

12. Thomson MC, Dalessandro U, Bennett S, Connor SJ, Langerock P, Jawara M, Todd J, Greenwood BM: **Malaria prevalence is inversely related to vector density in the Gambia, west-Africa**. *Trans Roy Soc Trop Med Hyg* 1994, **88**(6):638-643.

13. Hay SI, Rogers DJ, Toomer JF, Snow RW: **Annual Plasmodium falciparum entomological inoculation rates (EIR) across Africa: literature survey, internet access and review**. *Trans Roy Soc Trop Med Hyg* 2000, **94**(2):113-127.

14. Alonso P, Saute F, Aponte JJ, Gomez-Olive FX, Nhacolo A, Thomson R, Macete E, Abacassamo F, Ventura PJ, Bosch X *et al*: **Manhica DSS, Mozambique.** . In: *Population and health in developing countries: vol 1, population, health ad survival at INDEPTH sites.* edn. Ottawa: International Development Research Centre (IDRC); 2002: 189-195.

15. Shiff CJ, Minjas JN, Hall T, Hunt RH, Lyimo S, Davis JR: **Malaria infection potential of Anopheline mosquitos sampled by light trapping indoors in coastal Tanzanian villages**. *Med Vet Entomol* 1995, **9**(3):256-262.

16. Bejon P, Lusingu J, Olotu A, Leach A, Lievens M, Vekemans J, Mshamu S, Lang T, Gould J, Dubois M *et al*: **Efficacy of RTS,S/AS01E Vaccine against Malaria in Children 5 to 17 Months of Age**. *NEJM* 2008, **359**(24):2521-2532.

17. Owusu-Agyei S, Asante KP, Adjuik M, Adjei G, Awini E, Adams M, Newton S, Dosoo D, Dery D, Agyeman-Budu A *et al*: **Epidemiology of malaria in the forest-savanna transitional zone of Ghana**. *Malaria J* 2009, **8**.

18. Sylla EHK, Kun JFJ, Kremsner PG: **Mosquito distribution and entomological inoculation rates in three malaria-endemic areas in Gabon**. *Trans Roy Soc Trop Med Hyg* 2000, **94**(6):652-656.

19. Smith DL, Dushoff J, Snow RW, Hay SI: **The entomological inoculation rate and Plasmodium falciparum infection in African children**. *Nature* 2005, **438**(7067):492-495.

20. White MT, Bejon P, Olotu A, Griffin JT, Riley EM, Kester KE, Ockenhouse CF, Ghani AC: **The Relationship between RTS,S Vaccine-Induced Antibodies, CD4(+) T Cell Responses and Protection against Plasmodium falciparum Infection**. *PLOS One* 2013, **8**(4).

21. Spiegelhalter DJ, Best NG, Carlin BP, van der Linde A: **Bayesian measures of model complexity and fit (with discussion).** *J Royal Stat Soc B*, **64**(4):583-639
